# Sub-cellular population imaging tools reveal stable apical dendrites in hippocampal area CA3

**DOI:** 10.21203/rs.3.rs-2733660/v1

**Published:** 2023-04-21

**Authors:** Jason J Moore, Shannon K Rashid, Cara D. Johnson, Naomi Codrington, Dmitri B Chklovskii, Jayeeta Basu

**Affiliations:** 1 Neuroscience Institute, New York University Langone Health, New York, NY 10016, USA; 2 Center for Computational Neuroscience, Flatiron Institute, Simons Foundation, New York, NY 10010, USA; 3 Department of Neuroscience and Physiology, New York University Grossman School of Medicine, New York, NY 10016, USA; 4 Department of Psychiatry, New York University Grossman School of Medicine, New York, NY 10016, USA; 5 Center for Neural Science, New York University, New York, NY 10003, USA

## Abstract

Anatomically segregated apical and basal dendrites of pyramidal neurons receive functionally distinct inputs, but it is unknown if this results in compartment-level functional diversity during behavior. Here we imaged calcium signals from apical dendrites, soma, and basal dendrites of pyramidal neurons in area CA3 of mouse hippocampus during head-fixed navigation. To examine dendritic population activity, we developed computational tools to identify dendritic regions of interest and extract accurate fluorescence traces. We identified robust spatial tuning in apical and basal dendrites, similar to soma, though basal dendrites had reduced activity rates and place field widths. Across days, apical dendrites were more stable than soma or basal dendrites, resulting in better decoding of the animal’s position. These population-level dendritic differences may reflect functionally distinct input streams leading to different dendritic computations in CA3. These tools will facilitate future studies of signal transformations between cellular compartments and their relation to behavior.

## Introduction

Neurons receive distributed inputs onto their dendritic trees. These inputs are often arranged in a stratified manner, in which synapses from specific afferent brain areas arrive onto different dendritic compartments of a pyramidal cell. Broadly, dendrites of a prototypical pyramidal neuron are divided into apical and basal compartments. Beyond differences in the inputs they receive, apical and basal dendrites may differ in their molecular composition, structural and biophysical properties, and function^1^. It is known that dendrites support non-linear processing in the form of dendritic spikes^2–8^ and other ionic conductances^9,10^, which can tremendously enhance the computational capacity of individual neurons^11–15^ and support ensemble coding and plasticity^16–18^. However, it is unclear if the population activity of apical and basal dendrites differ in terms of content or stability across time.

Dendritic properties and computations have primarily been studied using single neuron recordings in brain slices or with calcium imaging^19,20^ in sparsely labeled *in vivo* preparations^21–25^. While these approaches allow faithful tracking of the activity of individual dendrites, they restrict experimental throughput, are often confined to only one dendritic compartment (apical or basal) and provide limited insight into the population dynamics of large numbers of dendrites. However, *in vivo* two-photon calcium imaging does allow simultaneous recording from large populations of neurons over long time periods. With stable implants and genetically expressed Ca^2+^ indicators, the same field of view can be revisited across several days. This allows experimenters to track the activity of the same neurons over time and quantify the stability or flexibility of response properties. Densely labeled preparations can yield hundreds to thousands of neurons, which enables population vector decoding of behavior^26,27^ and can yield insights into network properties and circuit-level function. Though calcium imaging provides subcellular resolution, thus far studies have not fully utilized the ability to track large populations of sub-cellular processes like dendrites over time.

A large factor limiting progress in understanding dendritic function *in vivo* is technical, particularly when recording from dense populations. Regions of interest (ROIs) belonging to individual neurons or sub-compartments of neurons must be identified, which can be tedious and time-consuming when done manually. While several software suites have been developed to automate this process when recording from fields of view composed of cell bodies^28–31^, dendrites’ diverse shapes makes morphology-based methods of dendritic ROI detection less reliable. To relate neural activity to behavior, the time-varying fluorescence values from each ROI must be estimated and transient periods of elevated activity identified. These calcium transients, or “events,” can then be used to quantify tuning properties. A simple approach of averaging values within ROIs is prone to contamination from overlapping ROIs, more so for dendrites compared to soma due to the smaller number of pixels per ROI. Human screening of detected transients can address these problems, but the amount of necessary manual labor and time will scale with the number of dendrites in a field of view, making manual checking infeasible. Overcoming such difficulties will tremendously expand the field’s ability to fully investigate sub-compartment level population activity and behaviorally relevant functional properties.

To address these challenges, we developed an automated detection algorithm flexible enough to identify dendritic and somatic ROIs in dense datasets. The algorithm identifies initial ROI estimates using minimal morphological assumptions and refines them using constrained non-negative matrix factorization (CNMF)^28,32^. It then screens putative calcium transients with an additional goodness-of-fit measure to eliminate spurious activity from undetected ROIs. To demonstrate the efficacy and utility of this approach, we applied it to several sets of data recorded from area CA3 of the mouse hippocampus^33^, an area containing “place cells” crucial for spatial navigation. Using this preparation, we characterized the spatial tuning properties of soma, apical dendrites, and basal dendrites during a simple navigation task. We find that while basal dendrites show lower event rates than soma, apical and basal dendrites have comparable spatial selectivity. However, by comparing activity across days in a familiar environment, we show that the apical dendrites have higher day-to-day stability than either basal dendrites or soma. This results in a better population decoding accuracy of position across days when recording from CA3 apical dendrites, indicating a functional divergence between sub-cellular compartments of hippocampal pyramidal neurons for long-term spatial representation.

## Results

### Dense dendritic fields of view are highly overlapping

**Extended Data Fig. 1 |:**
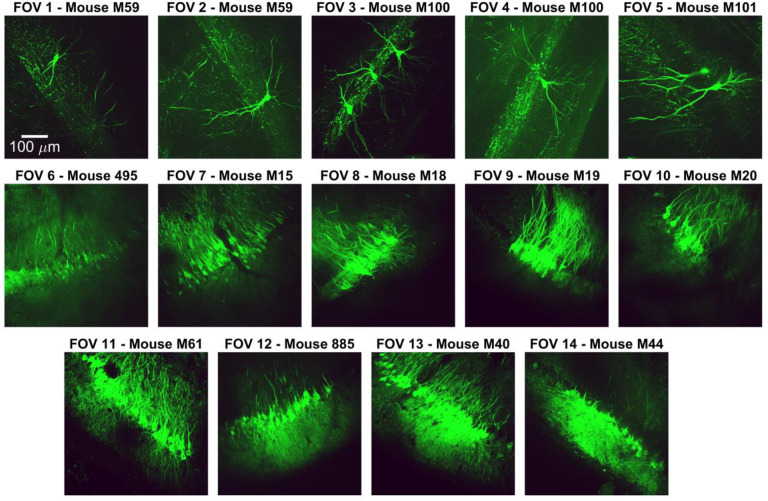
All fields of view. Maximum intensity projections of all fields of view used in the study. Fields of view are annotated with the mouse identification number each field of view was recorded from, as well as viral injection details for each. FOVs 7 and 11 were used in Figure 1c; FOV 2 was used in Figure 1d; FOV 9 was used in Figure 1e. FOVs 1–8, 10, and 11 were used in Figures 1f. FOVs 1, 2, and 6–14 were used in Figures 2, 3 and Extended Data Figure 2. FOVs 6–14 were used in Figure 4 and Extended Data Figure 4. FOVs 7–14 were used in Figures 5–7 and Extended Data Figures 5–10. Injection Parameters:Injections presented as Virus (Titer)FOV 1–2: AAV2.1-Syn-FLEX-GCaMP6f (8.2×10^12^); AAV2.1-CaMKII-Cre (1.36×10^8^).FOV 3–5: AAV2.1-Syn-FLEX-jGCaMP7b (1.64×10^12^); AAV2.1-CaMKII-Cre (2.44×10^9^).FOV 6: Transgenic GCaMP, GP5.5, no virusFOV 7–14: AAV2.1-CaMKII-GCaMP6f (2.76×10^13^)

**Fig. 1 |:**
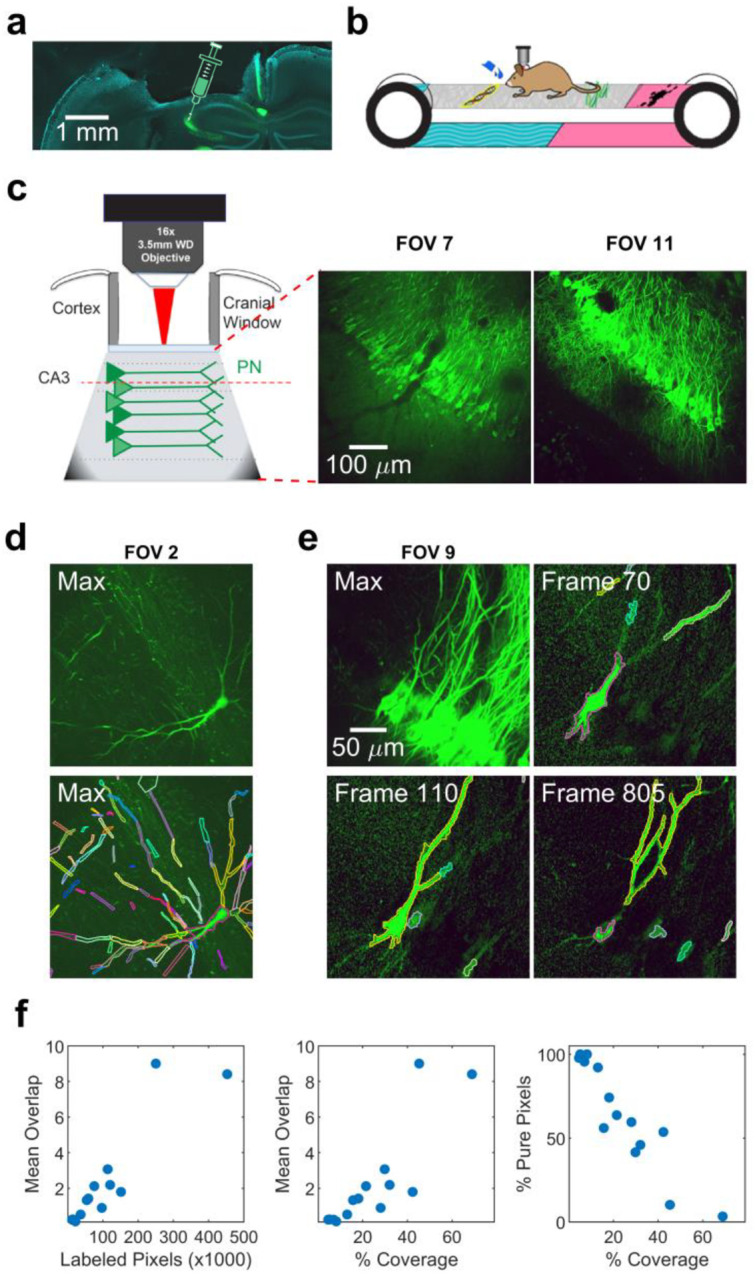
Sparse and dense GCaMP expression in hippocampal area CA3. **a**, Confocal image of coronal section of mouse hippocampus, showing site of viral injection and cranial window implant for imaging in area CA3. GCaMP+ CA3 pyramidal neurons are labeled in green. **b**, Top, experimental schematic of a mouse headfixed on a textured treadmill belt. **c**, Left, schematic of cranial window imaging setup. After aspirating the above cortex, a glass coverslip is secured above area CA3, allowing simultaneous imaging of cell bodies and dendrites. Right, maximum intensity projections of two sample fields of view (FOVs) in area CA3. **d**, Top, maximum intensity projection image of a sample sparse FOV. Bottom, manually labeled regions of interest (ROIs) overlaid on the maximum projection image. **e**, Top-left, maximum intensity projection image of a densely labeled FOV, with many overlapping dendrites. Other panels illustrate individual frames in the t-series in which dendritic and somatic ROIs can be clearly identified and labeled. **f**, Quantification of labeling density. As the number of identified ROIs (left) or percentage of pixels covered (middle) increased, the Mean Overlap increased. Right, as the coverage increased, the percentage of pixels belonging only to one ROI decreased.

To generate dendritic imaging data sets for developing and testing our algorithm, we expressed GCaMP6f or 7b in mice using a range of virus titers to control the labeling density (Fig. 1, Extended Data Fig. 1, see Methods)^19,21,33–35^. This allowed us to express GCaMP extremely sparsely to validate the basic mechanism of our algorithm, and then apply the same approaches to densely labeled datasets suitable for population level analysis. At low titers, single neurons were labeled in a single field of view (Fig. 1b). At higher titers the density of neurons and dendrites was so high that completely isolated soma or dendrites were quite rare. Most ROIs overlapped with at least one other ROI, and most pixels were covered by more than one ROI. To quantify the degree of overlap, human experts manually labeled these datasets, identifying groups of contiguous pixels with coordinated activity (Fig. 1c, see Methods). In the 8 mice with the highest titer virus injected, an average of 1300 ROIs/FOV were identified, covering 35% of the field of view (Fig. 1d). Manual labeling of these datasets took approximately 50 hours per field of view. On average, any given ROI at least partially overlapped with 3.6 other ROIs, and only 40% of pixels belonged to a single ROI, demonstrating a high degree of spatial overlap.

### Dendritic NMF algorithm enables efficient ROI extraction

The effort required to manually curate these datasets is massive, and the large amount of spatial overlap complicates efforts to accurately estimate activity. Hence, we sought a method to identify ROIs in an automated and unbiased way. CNMF^28,32^ has previously been used to identify ROIs and activities from partially overlapping neurons. The goal of CNMF is to identify ROIs and corresponding fluorescence traces that best approximate the original image stack. The objective function is the following:

$$\underset{A,C}{\text{argmin}}\|Y-AC{\|}_{F}^{2}+\eta \|C{\|}_{F}^{2}+\beta \|\text{sum}(A){\|}^{2};A\geq 0,C\geq 0$$

Here, ***Y*** represents the raw calcium imaging sequence, ***A*** represents the spatial footprints (ROIs), and ***C*** represents the fluorescence traces for each ROI. Background fluorescence from out-of-plane neuropil is accounted for by additional columns and rows in ***A*** and ***C***, respectively. The *η* and *β* terms are penalties to encourage sparsity in both the ROIs and fluorescence traces. This equation is solved by iteratively solving for ***A*** and ***C***, fixing one while optimizing the other (see Methods). This process can identify large neural populations but has not been systematically applied to densely labeled dendritic preparations.

**Fig. 2 |:**
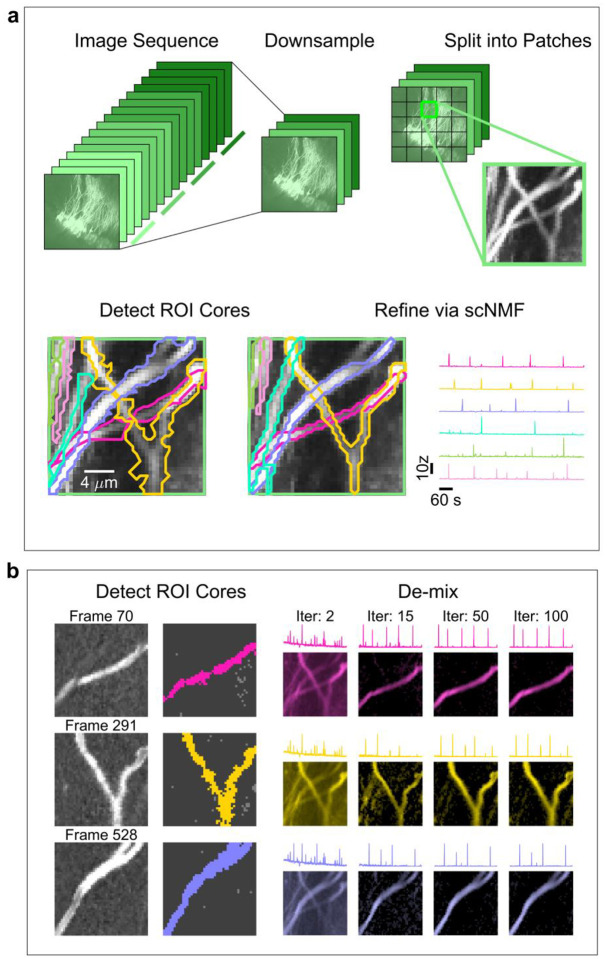
d-NMF pipeline. **a**, Pictorial representation of the workflow. Image sequences are first downsampled to reduce memory requirements. The downsampled image sequence is then split into patches. Within each patch, ROI cores are detected and their spatial footprint and temporal traces are iteratively estimated using sparse constrained NMF. Once all patches have been processed, overlapping ROIs from neighboring patches are tested to see if they can be merged. **b**, Algorithmic sketch of the ROI detection (left) and refinement (right). See Methods for further details. **c**, Top, illustration of accepting ROIs by skewness. Left, a true ROI (orange) and false ROI (brown). Scale bar = 15 μm. Right, the activity trace for the true ROI, with a high skewness value and the activity trace for the false ROI, with a low skewness value. Bottom left, ROC curves for individual FOVs (light gray) overlaid with the mean ROC (thick black), parametrized on the skewness cutoff of included ROIs. The mean False Positive Rate versus True Positive Rate are plotted for four example skewness cutoff values. Bottom right, classifier performance plotted against skewness cutoff. On average, a skewness cutoff of 3.8 resulted in optimal performance across all tested fields of view.

We developed a pipeline of unique initialization and refinement steps to be amenable to detecting soma and dendrites, which we call “dendritic NMF,” or d-NMF. We utilized temporal downsampling and patch-wise processing to reduce the memory and time requirements of the algorithm to enable processing on standard desktop computers (Fig. 2a, see Methods). Processing the image in patches had the added benefit of incorporating multiple background sources for each image patch, to better capture regional differences in neuropil signal. ROI initialization was performed by identifying contiguous regions of pixels active above a threshold (see Methods). Spatial footprints and temporal traces were then estimated by iterating through CNMF until convergence (Fig. 2b). ROIs were then thresholded and split into connected components (see Methods). These final morphological operations ensured that all identified ROIs were relatively compact and easily identifiable as single dendritic branches or soma.

The raw output of d-NMF contains any set of contiguous pixels that had simultaneously high activity at least once, which can result in identification of ROIs that do not clearly correspond to neural processes (false positives). In many brain regions, including the hippocampus, excitatory neural activity is characterized by long periods of quiescence punctuated by short periods of activity, resulting in a high skewness value. Hence, we quantified the skewness of the extracted calcium traces for each ROI (Fig. 2c), to allow clear removal of false positives while avoiding discarding real ROIs (false negatives).

**Extended Data Fig. 2 |:**
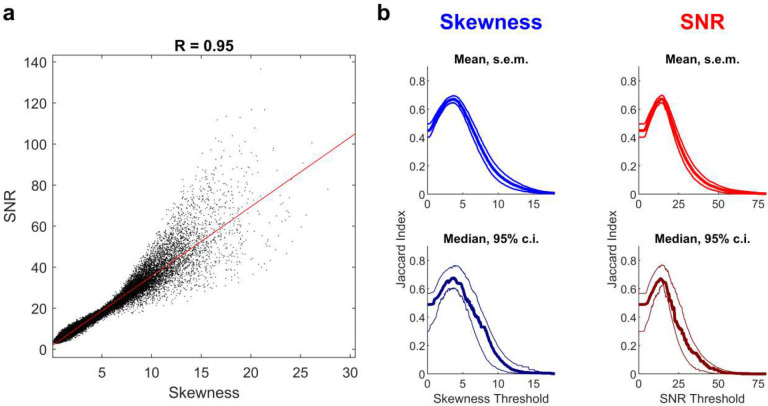
Skewness versus signal-to-noise ratio for ROI classification. **a**, Skewness is strongly correlated with signal-to-noise ratio (SNR), another measure of the quality of calcium traces (R=0.95, p=0, two-sided t-test, n=29,566 calcium traces from 11 fields of view from 10 mice). **b**, Overall performance of identifying true or false ROIs was similar when using Skewness (left, blue) or SNR (right, red). Peak +/− s.e.m. of mean Skewness: 0.67+/−0.02; Peak, 95% confidence interval of median Skewness: 0.68, [0.61, 0.76]; Peak +/− s.e.m. of mean SNR: 0.67 +/−0.03; Peak, 95% confidence interval of median SNR: 0.67, [0.62, 0.76]). n=11 FOVs for panel **b**.

**Extended Data Fig. 3 |:**
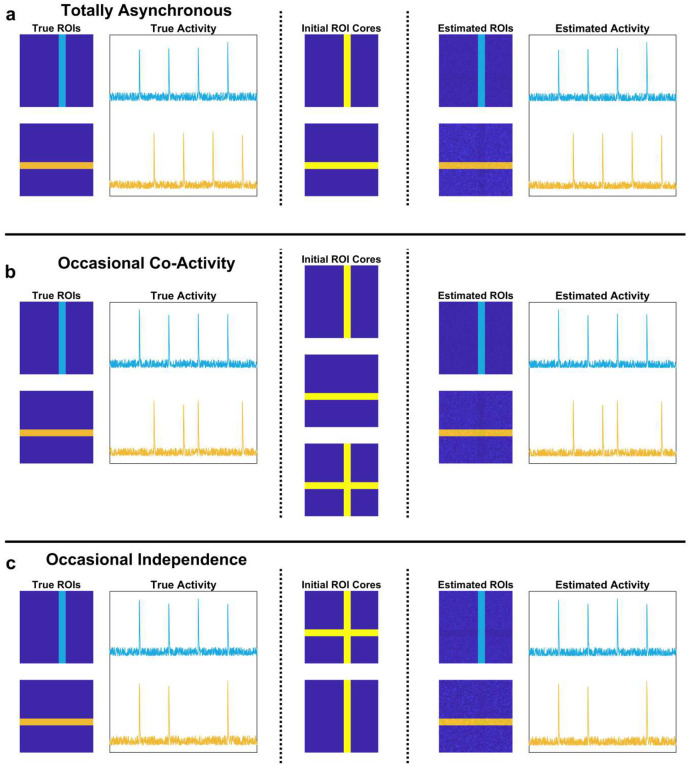
Example de-mixing. **a**, Example fiducial dataset demonstrating the ability of the algorithm to detect overlapping ROIs. Left, the true ROIs and activity are plotted. In this example, there are no time points at which the two ROIs, which overlap in space, are active at the same time. Middle, the initial detected ROI cores match the true ROIs. Right, after processing, both the estimated ROIs and estimated activity are nearly identical to the true ROIs and activity. **b**, Example situation where the two ROIs are mostly independent, but have a single calcium transient at the same time. The initial ROI cores reflect this, as a candidate ROI which is the union of the two is included. However, after the algorithm has converged, the only remaining ROIs left match the originals. **c**, Example situation where the two ROIs are mostly co-active, with a single calcium transient present in the top (vertical) ROI but not the bottom (horizontal) one. Since the horizontal ROI is never active on its own, it is not present in the initial ROI estimates. However, after processing, the surviving ROIs resemble the original two.

We explored the effect of varying the skewness cutoff by computing receiver operating characteristic (ROC) curves parametrized on skewness. To obtain an accurate measure of the false positive rate, all identified ROIs from the raw output of d-NMF were screened by a human expert and labeled valid or invalid based on morphology alone. The false positive and true positive rate of ROIs was evaluated using that labeling as ground truth. Across all samples, the mean area under the curve was 0.92, [0.90, 0.94], indicating skewness is a relatively strong separator of valid and invalid ROIs. Similar results were obtained using the signal-to-noise ratio (SNR) for classification (Extended Data Fig. 2). The optimal skewness value was relatively close across the population of fields of view, at 3.8, [3.2, 3.6], for both sparse and dense data. Note that this cutoff value may vary across brain regions, cell types, or fluorescent indicators. This process yields interpretable ROIs (Extended Data Fig. 3) of varying shape without random initialization or needing to specify the number of components to be detected beforehand.

### d-NMF matches manual labeling and exceeds existing methods

**Fig. 3 |:**
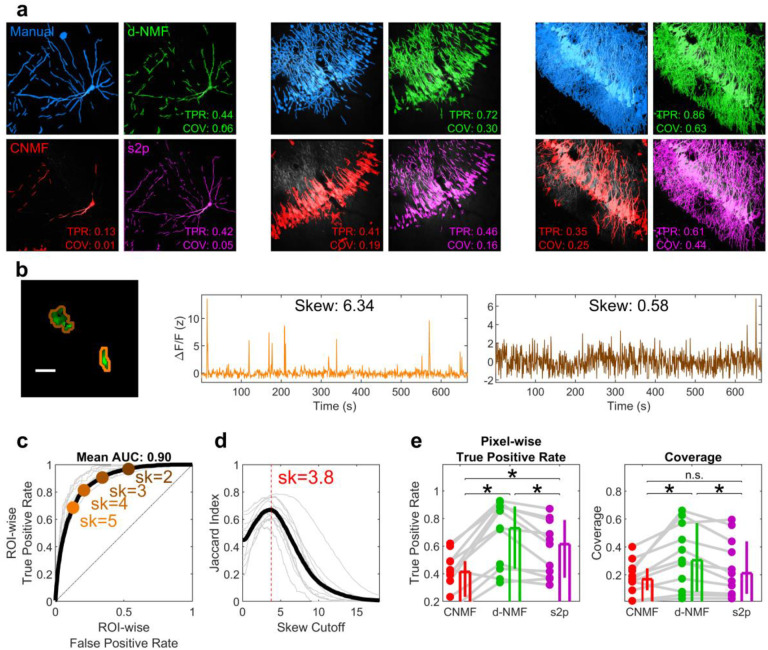
d-NMF accurately labels ROIS with highest coverage. **a**, 3 sample fields of view illustrating segmentation results on sparse, medium, and dense data. Color coded FOVs indicate ROIs identified manual segmentation (blue), d-NMF (green), CaImAn with default parameters (here labeled “CNMF”, red), and suite2p (magenta). **b**, The F1 score of identified pixels (compared to manual labeling) for d-NMF (green, 0.67, [0.52, 0.74]) was significantly higher (p=0.001) than that of CNMF (red, 0.46, [0.34, 0.51]), and significantly higher (p=0.007) than suite2p (s2p, purple: 0.59, [0.47, 0.74]). The F1 score of suite2p was also higher than CNMF (p=0.001). **c**, The True Positive Rate (TPR) of identified pixels (compared to manual labeling) for d-NMF (0.71, [0.44, 0.86]) was significantly higher (p=0.002) than that of CNMF (0.35, [0.21, 0.44]), and significantly higher (p=0.002) than suite2p (0.60, [0.37, 0.78]). **d**, d-NMF had significantly higher overall coverage than CNMF and suite2p (d-NMF Coverage: 0.27, [0.07, 0.56]; CNMF Coverage: 0.13, [0.08, 0.23]; suite2p Coverage: 0.21, [0.06, 0.43]; d-NMF vs CNMF: p=0.01; d-NMF vs suite2p: p=0.01; CNMF vs suite2p: p=0.003). n=11 FOVs, Wilcoxon sign-rank test for all. Values reported as median and 95% confidence interval of the median.

To demonstrate the accuracy and utility of d-NMF in detecting ROIs, we evaluated its performance with human-labeled ROIs used as ground truth. At a wide range of imaging densities, d-NMF closely matched manually drawn ROIs (Fig. 3). Because ROIs may be broken up into a different number of segments, labeling completeness was evaluated on a pixel-by-pixel basis, with true positives indicating that a pixel belongs to at least one ROI in both the manually-labeled and automatically labeled ROI sets. We evaluated performance using the F1 score (see Methods), which combines recall and precision, as human-labelers may miss ROIs that are not obvious, skewing false positive estimates.

We compared performance against CNMF, implemented with the CaImAn software package^28^, as well as another open-source analysis package suite2p^29^, (see Methods). The median F1 score using d-NMF was 0.67, exceeding that of CNMF (0.46) and suite2p (0.59) (Fig. 3b, Left). Similarly, the True Positive Rate of d-NMF (0.71) exceeded CNMF (0.35) and suite2p (0.60) (Fig. 3c). The percentage of the FOV covered by ROIs (coverage) using d-NMF (27%) was also higher than CNMF (13%) and suite2p (21%) (Fig. 3d, Right). In particular, CaImAn missed several ROIs in dense regions of the FOV. While suite2p performed comparably with d-NMF in sparse preparations (Fig. 3a, Left), dense preparations were more completely labeled using d-NMF (Fig. 3a, Right). d-NMF performed well on both sparse and dense datasets with a single set of parameters, whereas other methods may need careful parameter tuning depending on the density of the preparation.

### A fitness trace to refine activity estimates and eliminate cross contamination

Densely labeled datasets present added difficulties in estimating the fluorescence activity of each ROI due to overlapping sources. Given a complete set of ROIs, CNMF accurately de-mixes these signals and properly assigns activity to each ROI. However, despite the best efforts of manual labeling or automated labeling, some ROIs will go undetected. This will lead to “contamination” of signals from identified ROIs by unidentified ones. Such contamination can affect estimates of activity rates, tuning properties, stability, or correlation with related ROIs. While simply setting a high threshold for transient detection may reliably screen out false transients, this runs the risk of excluding low-amplitude calcium transients which may represent different firing modes.

**Fig. 4 |:**
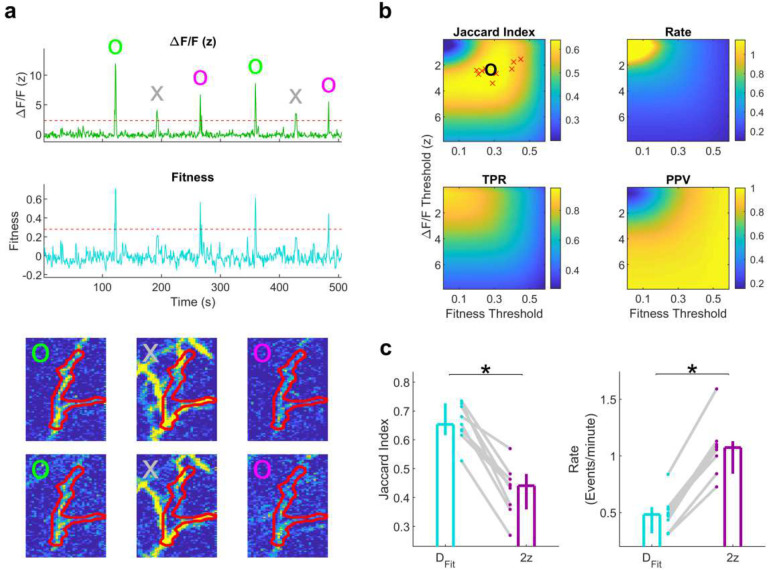
Fitness trace screens out false activity. **a**, Top, raw fluorescence trace (z-scored) of the ROI shown below. Example large amplitude true events, small amplitude true events, and false events are marked in green circles, magenta circles, and gray crosses, respectively. Middle, the Fitness Trace computed from the above fluorescence trace. Bottom, frames of large amplitude true events (green circles), false events (gray crosses), and small amplitude true events (magenta circles). **b**, Classification performance, as measured by the Jaccard Index, plotted as a function of threshold on ΔF/F and Fitness Trace (see Methods). The global peak is indicated with a black circle, and peaks for individual FOVs are marked with red crosses. **c**, Left, classifier performance for DNMF with Fitness Trace (D_Fit_) compared to a simpler detection method (2z, see Methods). D_Fit_: 0.65, [0.62, 0.73]; 2z: 0.44, [0.36, 0.48]; D_Fit_ vs 2z: p=3.9×10^−3^. Right, the detected event rate for the same 2 methods, illustrating the importance of correct classification. D_Fit_: 0.48, [0.32, 0.55] Events/minute; 2z: 1.07, [0.84, 1.13] Events/minute; n=9 sessions for all. D_Fit_ vs 2z: p=3.9×10^−3^. N=9 FOVs, Wilcoxon sign-rank test for all. Values reported as median and 95% confidence interval of the median.

To both evaluate the degree of contamination and to provide an additional tool to correct this, we developed the concept of a “Fitness Trace” for each ROI (Fig. 4a), defined as the frame-by-frame correlation between the spatial footprint of that ROI with the video (see Methods). The intuition behind this trace is that true activity in a given ROI occurs when all pixels show elevated activity, not just some. Significant calcium transients were detected by thresholding the Fitness trace along with the original calcium trace (Fig. 4b). Overall classifier performance was evaluated by computing the Jaccard index (the size of the intersection divided by the size of the union)^36^ between detected events and manually labeled true events (see Methods). Across 9 different FOVs there was a relatively consistent optimal parameter range, at a ΔF/F threshold of 2.3z and a Fitness threshold of 0.28.

**Extended Data Fig. 4 |:**
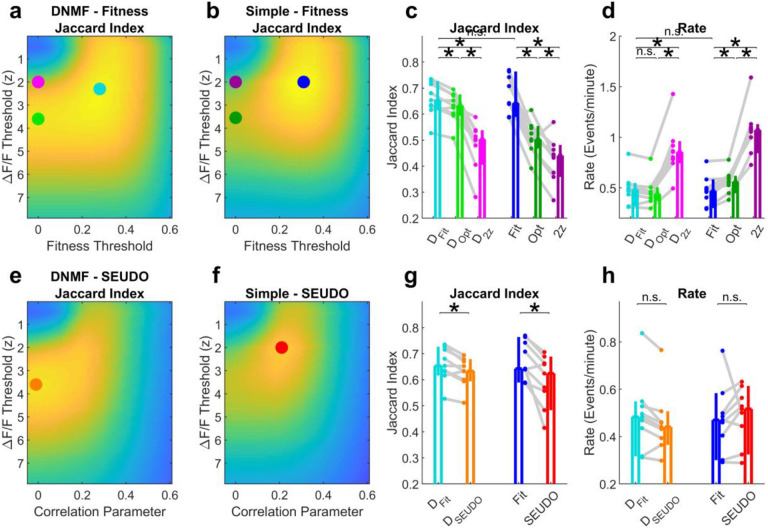
Event detection for different methods. **a**, Classifier performance for different event classification methods utilizing the de-mixed fluorescence trace and the fitness trace (see Methods). **b**, Classifier performance for different event classification methods utilizing the simple fluorescence trace (see Methods) and the fitness trace. **c**, D_Fit_ outperformed other methods that did not use the fitness trace. **d**, Same as **c** but for event rate. **e**, Classifier performance when using the SEUDO correlation parameter instead of the Fitness trace on top of the de-mixed fluorescence trace. **f**, Classifier performance when using the SEUDO correlation parameter instead of the Fitness trace on top of the simple fluorescence trace. **g**, Overall classification performance was slightly but significantly higher using the Fitness trace than using SEUDO, both for de-mixed data and simple data. **h**, Detected event rates were not different across the different methods. See Table 1 for summary statistics.

We compared detection accuracy and the rate of detected transients from our optimized Fitness method to those of other event detection methods (Fig. 4c). A widely used approach is to set a threshold of 2 standard deviations above the mean on the raw ΔF/F traces, with no de-mixing (with an additional false positive rejection criterion, see Methods). We call this method “2z.” We call our method using optimal values for the ΔF/F threshold and Fitness threshold “D_Fit_.” Event classification performance was significantly higher using D_Fit_ (0.65) compared to 2z (0.48). Other detection methods incorporating different elements of these two approaches achieved intermediate results (Extended Data Fig. 4). The use of the Fitness Trace also was comparable to or exceeded other related measures^37^ (Extended Data Fig. 4e–h). More striking, the estimated calcium transient rate differed by nearly a factor of 2 when comparing the two methods (Fig. 4c). This demonstrates that accurate event detection and screening is a vital step in processing data from densely labeled FOVs.

### Spatial coding properties of dendrites in CA3

**Fig. 5 |:**
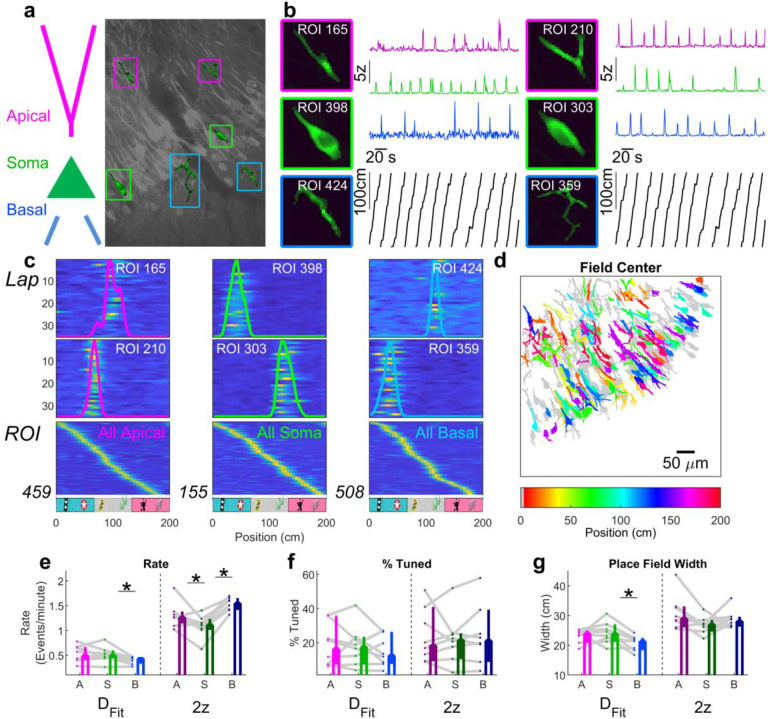
CA3 dendritic spatial coding properties. **a**, Left, Schematic designating the apical dendrites, soma, and basal dendrites. Right, field of view with two each of apical dendrites (magenta), soma (green), and basal dendrites (blue) highlighted. **b**, Expanded view of sample apical dendritic, somatic, and basal dendritic ROIs with activity across time in corresponding colos. Position of the mouse is plotted in black. **c**, Top, middle, for the sample ROIs, ΔF/F is plotted against lap number and position. The trial-averaged tuning curve is overlaid for each ROI. Bottom, all statistically significant tuning curves for apical, somatic, and basal ROIs across all mice, demonstrating a relatively uniform tiling of the environment in each population. **d**, Sample FOV color-coded according to the position of peak activity for each ROI. **e**, When estimated using the D_Fit_ procedure, basal dendrites had a significantly lower mean transient rate (0.44, [0.32, 0.46] Events/minute) than the soma (0.50, [0.44, 0.60] Events/minute; p=0.039, Wilcoxon sign-rank test, n=8 sessions). This difference was similar in respect to apical dendrites (0.50, [0.40, 0.67] Events/minute), but did not reach statistical significance (p=0.055, Wilcoxon sign-rank test). Apical dendrite event rate was not significantly different than somatic event rate (p=1.0, Wilcoxon sign-rank test). If estimated using the 2z procedure, both apical and basal dendrites had higher event rates than soma, an artifact of contaminated activity from overlapping ROIs (Apical: 1.3, [1.1, 1.4] Events/minute; Soma: 1.1, [1.0, 1.2] Events/minute; Basal: 1.6, [1.4, 1.7] Events/minute; Apical vs Soma: p=0.039; Apical vs Basal: p=0.05; Soma vs Basal: p=7.8×10^−3^, Wilcoxon sign-rank test for all). **f**, The percentage of ROIs that were tuned were similar across all 3 ROI types regardless of the detection method used. D_Fit_: Apical: 16, [7, 36]%; Soma: 18, [7, 23]%; Basal: 13 [7, 26]%; apical vs soma, p=0.95; basal vs soma, p=0.31; apical vs basal, p=0.55. 2z: Apical: 18, [9, 41]%; Soma: 22, [10, 25]%; Basal: 21, [9, 39]%; apical vs soma, p=0.95; basal vs soma, p=0.38; apical vs basal, p=0.84. **g**, For ROIs with statistically significant tuning, the width of place fields was slightly lower for basal dendrites (21, [18, 23] cm) compared to soma (24, [21, 27] cm) or apical dendrites (24, [21, 25] cm) when quantified using D_Fit_, though the effect was only significant between basal dendrites and soma (basal vs soma, p=0.039, Wilcoxon sign-rank test; apical vs basal, p=0.11, Wilcoxon sign-rank test, apical vs soma, p=0.38, Wilcoxon sign-rank test). There was no difference across compartments when field width was quantified using the 2z procedure (Apical: 29, [26, 33] cm; Soma: 27, [25, 28] cm; Basal: 28, [26, 30] cm; apical vs soma, p=0.055; basal vs soma, p=0.31; apical vs basal, p=0.74). Values reported as median and 95% confidence interval of the median.

To demonstrate the utility of d-NMF, we applied it to dense fields of view obtained in area CA3 of the mouse hippocampus, as mice performed a random foraging task on a textured belt (Fig. 5, see Methods). Somata of pyramidal cells in area CA3 demonstrate place coding, where they have reliable elevated activity levels in restricted regions of space. Using d-NMF, across 8 mice we identified 794 total somatic ROIs, 2910 apical dendritic ROIs, and 4577 basal dendritic ROIs. This allowed us to examine and compare the activity and spatial tuning properties of populations of apical and basal dendrites.

**Extended Data Fig. 5 |:**
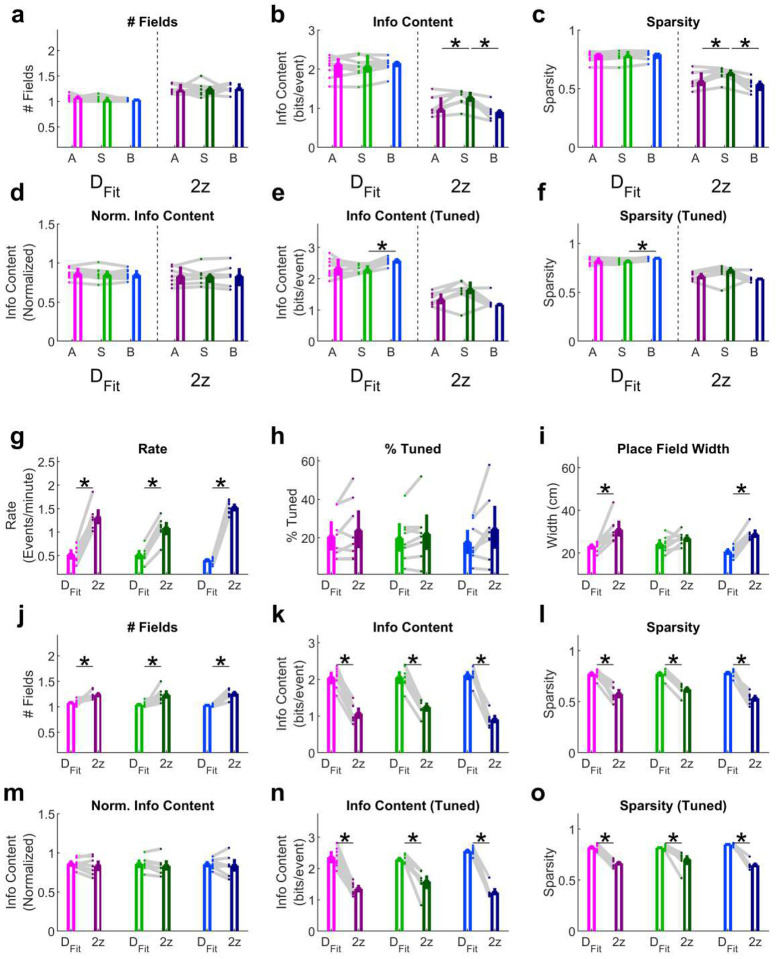
Additional quantification of basic place tuning properties. See Tables 2–3 for summary statistics.

**Extended Data Fig. 6 |:**
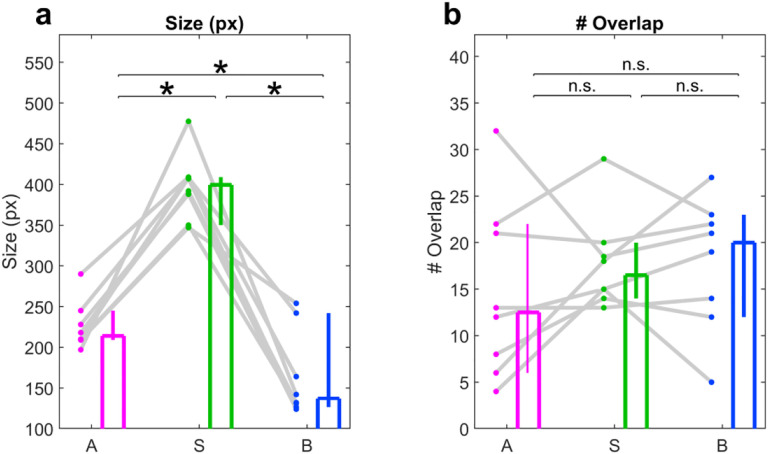
ROI size and overlap number across compartments. **a**, The mean ROI size (in pixels), was greatest for soma (400, [350, 409] pixels), followed by apical dendrites (215, [210, 245] pixels), then basal dendrites (135, [125, 240] pixels). Apical vs Soma: p=7.8×10^−3^; Apical vs Basal: p=0.04; Soma vs Basal: p=7.8×10^−3^. **b**, The mean number of overlapping ROIs was not significantly different between apical dendrites (12.5, [6, 22]), soma (16.5, [14 20]), or basal dendrites (20, [12, 23]). Apical vs Soma: p=0.38; Apical vs Basal: p=0.38; Soma vs Basal: p=0.87. n = 8 FOVs, Wilcoxon sign-rank test used for all statistics.

**Extended Data Fig. 7 |:**
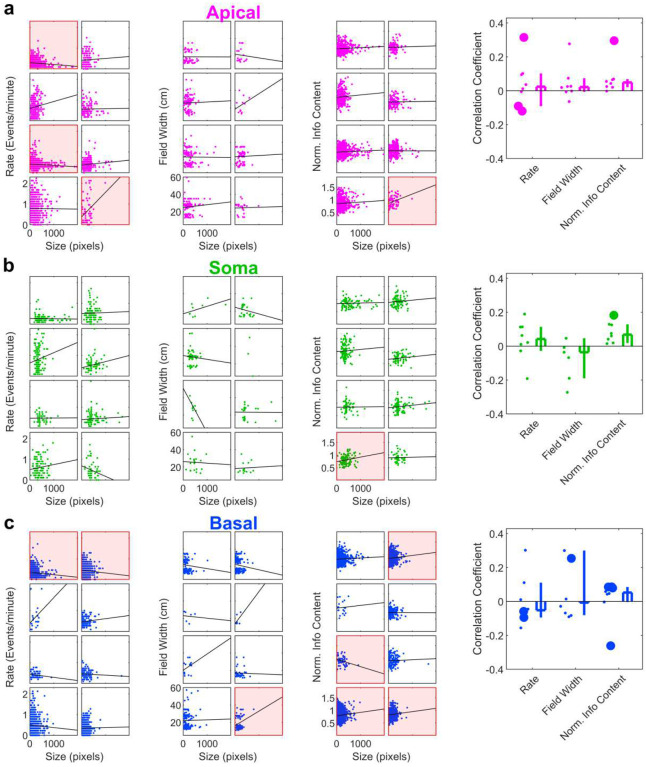
Relationship between ROI size and basic place tuning properties. **a**, Left, scatter plots relating Apical Dendrite ROI size (in pixels) to Event Rate, Field Width, and Normalized Information Content. Each box contains data from an individual mouse. Datasets with a statistically significant correlation are highlighted in red. Right, the distribution of correlation coefficients across all mice. Significant correlations in individual mice are marked with large dots. Rate: 0.02, [−0.09, 0.10]; Field Width: 0.02, [−0.01, 0.07], Norm. Info Content: 0.05, [0.02, 0.07]. n = 8 FOVs for all. **b**, Same as **a** but for somatic ROIs. Rate: 0.04, [−0.03, 0.11], n=8 FOVs; Field Width: −0.04, [−0.19, 0.05], n=7 FOVs, Norm. Info Content: 0.07, [0.02, 0.13], n=8 FOVs. **c**, Same as **a** but for basal dendrite ROIs. Rate: −0.05, [−0.09, 0.11]; Field Width: −0.01, [−0.08, 0.30]; Norm. Info Content: 0.05, [−0.003, 0.09], n=8 FOVs for all.

**Extended Data Fig. 8 |:**
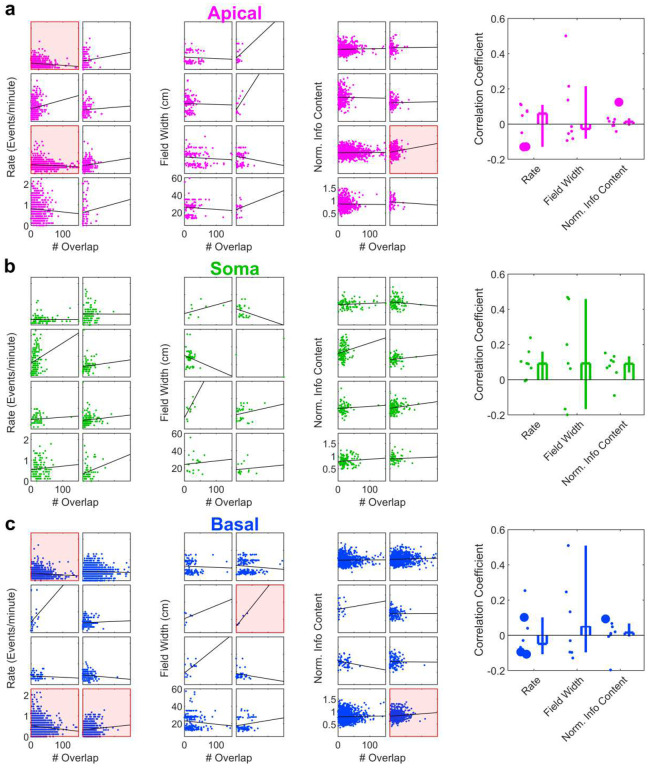
Relationship between overlap number and basic place tuning properties. **a**, Left, scatter plots relating Apical Dendrite ROI overlap count to Event Rate, Field Width, and Normalized Information Content. Each box contains data from an individual mouse. Datasets with a statistically significant correlation are highlighted in red. Right, the distribution of correlation coefficients across all mice. Significant correlations in individual mice are marked with large dots. Rate: 0.06, [−0.13, 0.11]; Field Width: −0.03, [−0.08, 0.22], Norm. Info Content: 0.01, [−0.01, 0.03]. n = 8 FOVs for all. **b**, Same as **a** but for somatic ROIs. Rate: 0.09, [−0.001, 0.16], n=8 FOVs; Field Width: 0.09, [−0.17, 0.46], n=7 FOVs, Norm. Info Content: 0.09, [0.04, 0.13], n=8 FOVs. **c**, Same as **a** but for basal dendrite ROIs. Rate: −0.05, [−0.11, 0.10]; Field Width: 0.05, [−0.10, 0.51]; Norm. Info Content: 0.02, [−0.01, 0.07], n=8 FOVs for all.

While spatial tuning in CA3 PN somata has been well described through electrophysiology approaches^38–40^ and more recently with Ca^2+^ imaging^41,42^, we know little about how reliably CA3 dendrites encode place and their spatial tuning properties. Using our automated approaches, we identified spatially tuned CA3 dendrites (“place dendrites”) both in the apical and basal compartments (Fig. 5a–d). Overall activity rates and spatial tuning properties were quite similar between soma and dendrites, with basal dendrites having a lower event rate than soma (Fig. 5e). A similar percentage of ROIs were significantly spatially tuned (Fig. 5f). The place field width of tuned apical dendrites and soma were largely similar (Fig. 5g), while basal dendrite place field widths were significantly smaller than soma. Other spatial tuning properties such as the number of place fields, information content, and sparsity were also similar across compartments (Extended Data Fig. 5). Basic spatial tuning characteristics were not systematically related to the ROI size (Extended Data Figs. 6, 7) or the number of overlapping ROIs (Extended Data Fig. 8).

The above tuning properties were computed using events detected using the D_Fit_ procedure. We re-computed spatial tuning properties using the more traditional 2z detection method, which overestimated the event rate, place field width, and number of place fields (Extended Data Fig. 5), and reported a significantly lower firing rate in the soma compared to dendrites (Fig. 5e). This underscores the importance of proper event detection and illustrates that scientific findings can be affected by the choice of analysis method.

### Apical dendrites are more stable across days and provide better place decoding

**Extended Data Fig. 9 |:**
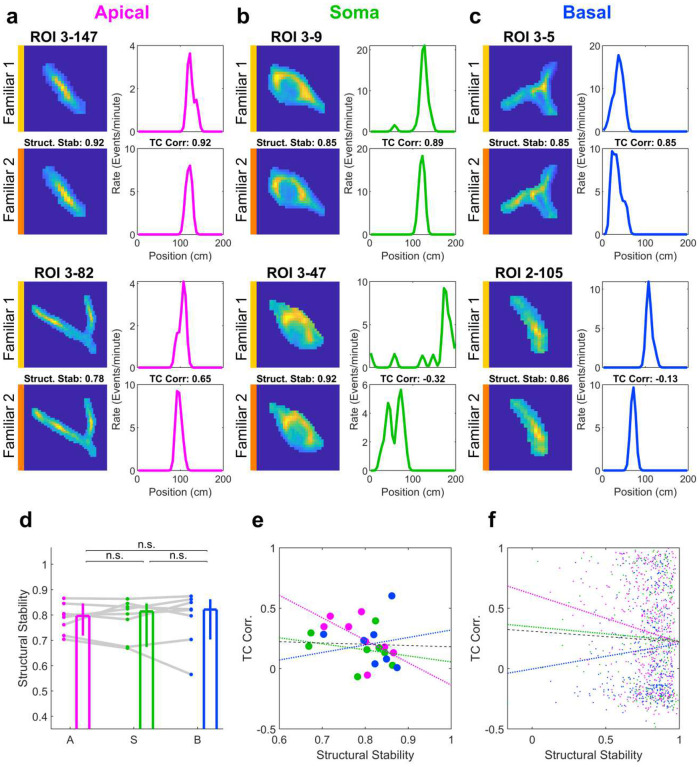
Stability of FOV and ROIs across days. **a**, Two example apical dendrite ROIs tracked across consecutive days: Familiar 1 (top, yellow bar) and Familiar 2 (bottom, orange bar). The surface plots on the left indicate the mean value of the ROI across the frames with detected calcium transients. Structural stability is defined as the correlation coefficient between these two surface plots. The plots on the right indicate the rate map of the given ROI as a function of position. Tuning Curve (TC) correlation is defined as the correlation coefficient between these two rate maps. **b**, Same as **a** but for two example somatic ROIs. Note the second example has high structural stability but an entirely different tuning curve between the two days. **c**, Same as **a** but for two example basal dendrite ROIs. As in **b**, note the second example has high structural stability but low TC correlation. **d**, Structural stability was relatively high and not statistically significantly different across compartment types (Apical: 0.80, [0.72, 0.85]; Soma: 0.81, [0.67 0.85]; Basal: 0.82, [0.70, 0.86]; Apical vs Soma: p=0.74; Apical vs Basal: p=0.64; Soma vs Basal: p=0.74, Wilcoxon sign-rank test, n=8 FOVs for all). **e**, There was no systematic relationship between structural stability and across-day TC correlation when averaged within mice (Effect of structural stability on TC corr: p=0.29; Effect of interaction between compartment type and structural stability: p=0.18, 2-way ANOVA, n = 8 sessions * 3 compartment types). **f**, There was no systematic relationship between structural stability and across-day TC correlation when datapoints were considered independent (Effect of structural stability on TC corr: p=0.20), though there was an effect of interaction (Effect of interaction between compartment type and structural stability: p=0.005, 2-way ANOVA, n=1051 ROIs).

**Extended Data Fig. 10 |:**
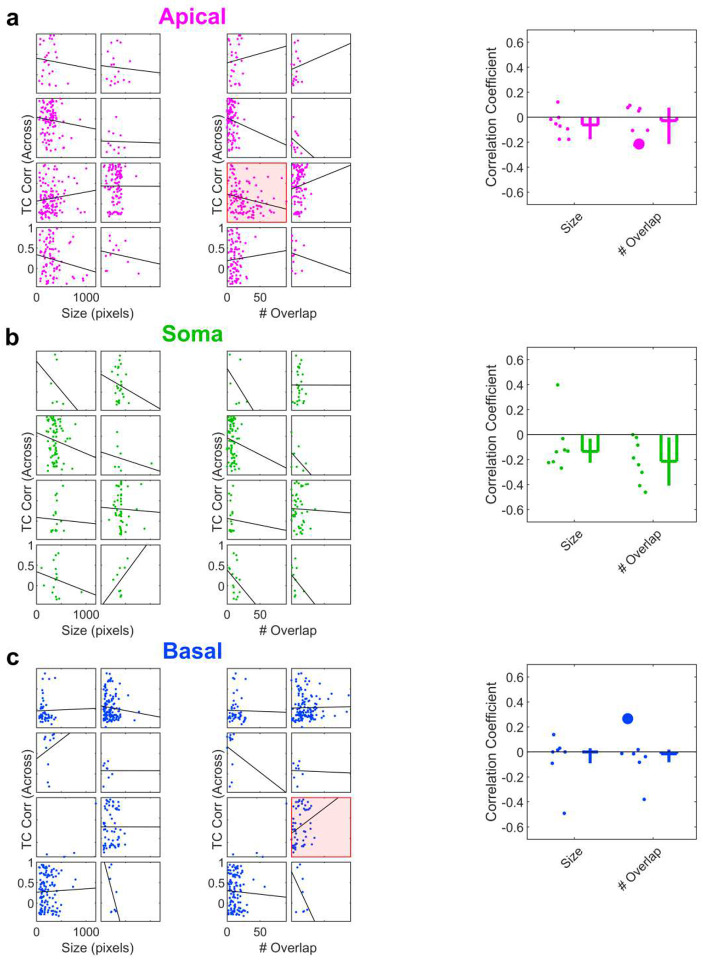
Relationship between size and overlap number with across-day tuning curve correlation. **a**, Left, scatter plots relating Apical Dendrite ROI overlap count and ROI size to across-day Tuning Curve correlation. Each box contains data from an individual mouse. Datasets with a statistically significant correlation are highlighted in red. Right, the distribution of correlation coefficients across all mice. Significant correlations in individual mice are marked with large dots. Size: −0.06, [−0.18, −0.001]; # Overlap: −0.03, [−0.21, 0.08]. n = 8 FOVs for all. **b**, Same as **a** but for somatic ROIs. Size: −0.14, [−0.22, 0.03], n=7 FOVs; # Overlap: −0.21, [−0.41, −0.04], n=7 FOVs. **c**, Same as **a** but for basal dendrite ROIs. Size: −0.0004, [−0.09, 0.03], n=8 FOVs; # Overlap: −0.02, [−0.08, 0.02], n=8 FOVs.

**Fig. 6 |:**
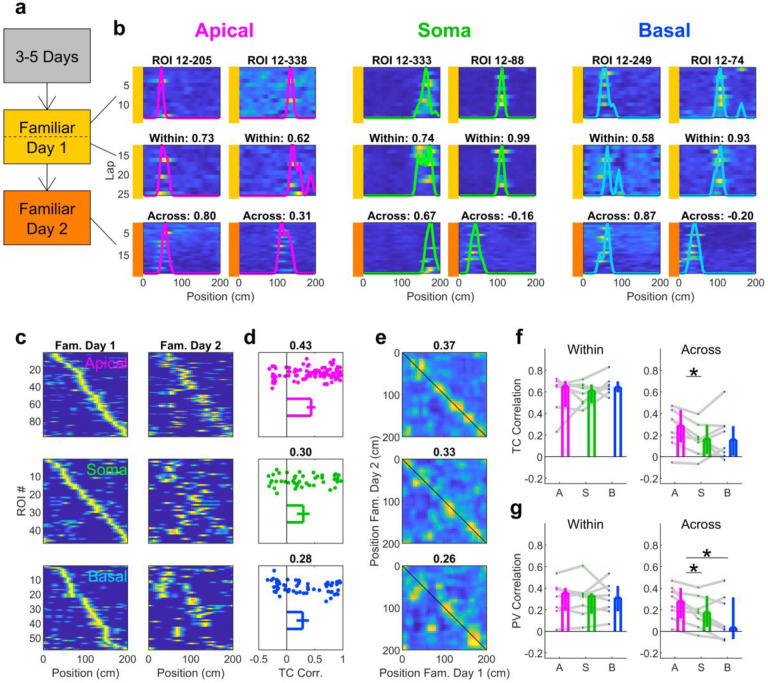
Stability measures across compartments show apical dendrites are more stable across days. **a**, Experimental timeline schematic. Mice ran on a treadmill belt for 3–5 days, after which the environment was designated as “familiar.” ROIs were tracked over the next two days, designated as Familiar Day 1 (yellow) and Familiar Day 2 (orange). **b**, Example apical, somatic, and basal ROIs demonstrating different short- and long-term stability. Each column represents a different ROI. The top row shows the raw activity and rate map for the first half of Familiar Day 1. The middle row shows the same for the second half of Familiar Day 1. The bottom row shows the same for Familiar Day 2. “Within” measures are the tuning curve correlation between Familiar Day 1 First and Second Half. “Across” measures are the tuning curve correlation between Familiar Day 1 and Familiar Day 2. **c**, Rate maps for all apical, soma, and basal ROIs in a single FOV on Familiar Day 1 (left) and Familiar Day 2 (right). ROIs are sorted according to the position of the peak of their tuning curve in Familiar Day 1, and that order is maintained for Familiar Day 2. **d**, Distributions of the across-day tuning curve correlations for the data shown in (C). Apical dendrites had a generally higher correlation (0.43, [0.35, 0.51], n=98 apical ROIs) than soma (0.29, [0.18, 0.41], n=47 somatic ROIs) or basal dendrites (0.28, [0.17, 0.39], n=58 basal ROIs). **e**, Population vector overlap matrices for the data shown in (C). The mean value across the diagonal is displayed in the title for each compartment type. **f**, Left, within-day tuning curve correlations were not different across compartment type (apical: 0.65, [0.46, 0.70]; soma: 0.61, [0.49, 0.67]; basal: 0.64, [0.60, 0.70]; apical vs soma: p=0.74; basal vs soma: p=0.20; apical vs basal: 0.64; n=8 sessions, Wilcoxon sign-rank test for all). Right, in contrast, apical dendrites had higher across-day TC correlations (0.29, [0.13, 0.43]) compared to soma (0.16, [0.03, 0.30]) and basal dendrites (0.16, [0.01, 0.28]), though this effect was only statistically significant between apical dendrites and soma (apical vs soma, p=7.8×10^−3^; basal vs soma, p=0.84; apical vs basal: p=0.31, Wilcoxon sign-rank test for all). **g**, Left, within-day population vector correlations were not significantly different across compartments (apical: 0.36, [0.19, 0.40]; soma: 0.33, [0.16, 0.35]; basal: 0.31, [0.19, 0.42]; apical vs soma: p=0.64; basal vs soma: p=0.84; apical vs basal: 0.84; n=8 sessions, Wilcoxon sign-rank test for all). Right, apical dendrites had higher across-day PV correlations (0.28, [0.12, 0.41]) compared to soma (0.28, [0.04, 0.31]) and basal dendrites (0.03, [−0.07, 0.32]), which were not significantly different from each other (apical vs soma: p=7.8×10^−3^; apical vs basal: p=0.039; basal vs soma: p=0.15; n=8 sessions, Wilcoxon sign-rank test for all). Values reported as median and 95% confidence interval of the median.

An advantage of optical recording techniques is the ability to track the same ROIs across multiple days. Thus, we quantified the short-term (within-day) and long-term (across-day) stability of apical dendrites, soma, and basal dendrites in a familiar environment (Fig. 6). Within-day stability was not significantly different across compartments whether quantified using either Tuning Curve (TC) correlation or Population Vector (PV) correlation (see Methods). However, apical dendrites were more stable across days compared to soma (either using TC or PV correlation) and basal dendrites (using PV correlation). The differences in across-day stability were not due to fields of view distorting across days (Extended Data Fig. 9), and could not be attributed to differences in ROI size or overlap (Extended Data Fig. 10).

**Fig. 7 |:**
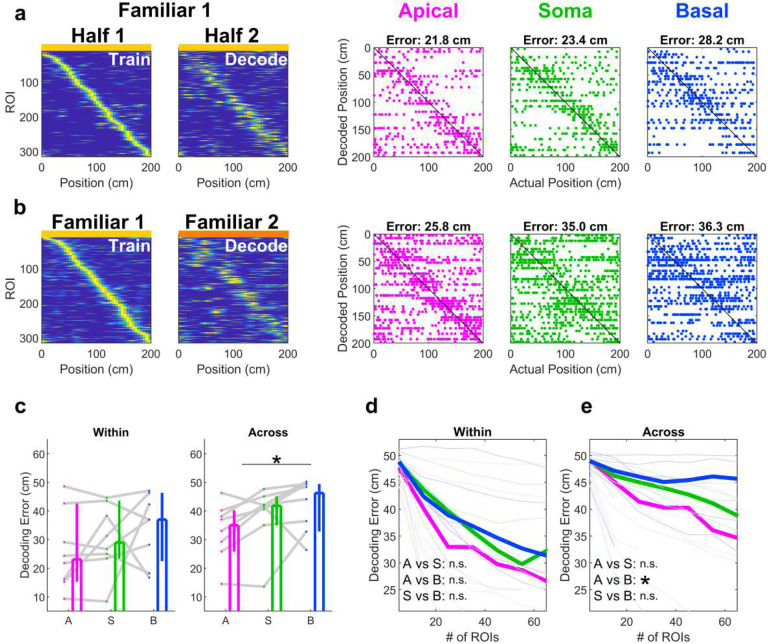
Population vector decoding for apical dendrites outperforms basal dendrites. **a**, Left, sorted rate maps of all ROIs from a single FOV in the first half and second half of Familiar Day 1. The maps from Half 1 were used to train a decoder to decode the position in Half 2. Right, example decoded position for the same FOV using only apical, somatic, or basal ROIs. **b**, Same as in **a** except data from the entire Familiar Day 1 was used to train a model to decode Familiar Day 2. Note that the error is generally larger compared to within-day decoding, and that apical dendrites show the lowest decoding error. **c**, Left, within-day decoding error was not significantly different across compartment types (apical: 23, [15, 41] cm; soma: 29, [23, 44] cm; basal: 37, [18, 46] cm; apical vs soma: p=0.64; basal vs soma: p=0.64; apical vs basal: p=0.25, N = 8 sessions, Wilcoxon sign-rank test for all). Right, across-day decoding error was lower for apical dendrites (35, [26, 40] cm) compared to soma (42, [35, 45] cm) or basal dendrites (46, [33, 49] cm), but the difference was only statistically significant between apical dendrites and basal dendrites (apical vs basal: p=0.016; apical vs soma: p=0.055; basal vs soma: p=0.15; n=8 sessions, Wilcoxon sign-rank test for all). **d**, There was no statistically significant effect of compartment on within-day decoding error when controlling for the number of ROIs used to decode position (A vs S: p=0.75; A vs B: p=0.62; S vs B: p=0.41; two-way ANOVA, see Methods). **e**, The differences across compartments in across-day decoding error persisted after controlling for the number of ROIs used to decode position (A vs S: p=0.96; A vs B: p=0.04; S vs B: p=0.08; two-way ANOVA, see Methods). Values reported as median and 95% confidence interval of the median.

Because apical dendrites showed better across-day stability compared to soma and basal dendrites, we predicted that apical dendrites would also outperform the other compartments in population vector decoding. Thus, we constructed population vector decoders using only apical dendrites, soma, or basal dendrites, and tested their ability to decode position within a session (Fig. 7a) or across sessions (Fig. 7b). There was no significant difference between decoding accuracy within-day. However, apical dendrites had significantly lower decoding error compared to basal dendrites across days (Fig. 7c). Population vector decoding is known to be highly dependent on the number of sources used to train the model, so we repeated the analysis using random subsets of data. Consistently, we found no differences across groups for within-day decoding (Fig. 7d), but a significant effect of compartment group on across-day decoding, with apical dendrites showing significantly better decoding accuracy compared to basal dendrites (Fig. 7e).

## Discussion

In summary, we developed an algorithm and toolkit that facilitates analysis of densely labeled fields of view containing both apical and basal dendrites and cell bodies (Fig. 1), enabling large-throughput *in vivo* investigation of dendritic activity. The algorithm utilizes the well-validated mathematical framework of CNMF while providing automated initialization flexible enough to identify neural processes of varied shape and size (Figs. 2, 3). The addition of the Fitness Trace post-hoc helps screen out false calcium transients to more accurately estimate dendritic tuning properties (Fig. 4). Using these tools, we demonstrate spatial tuning in apical and basal dendrites of pyramidal neurons in CA3 (Fig. 5). While the proportion of tuned ROIs and within-day tuning properties were largely comparable across all compartments, basal dendrites showed lower event rates and place field width than soma. By comparing longer term population dynamics across days, we demonstrate that apical dendrites are more stable than soma or basal dendrites across-days (Fig. 6). This leads to better accuracy when constructing a population vector decoder to predict the animal’s position (Fig. 7). These population-level analyses would not be possible with sparsely labeled preparations, and we were only able to take advantage of our densely labeled preparations using the analytical tools we have developed.

Densely labeled dendritic fields of view present a number of challenges for existing automated detection methods, particularly the initialization step. Initialization is often based on morphology, with parameters to be set for the expected diameter of an ROI. This assumes a uniform size of circular ROIs throughout the FOV, which is not the case for dendrites. Random initialization does not suffer from the drawbacks of morphology-based approaches, but there is no guarantee of complete labeling or consistent labeling across iterations. Additionally, the number of components must be specified for initialization, which may vary over the field of view. One approach is to over-specify the model and then discard unusable components afterwards^32^, but this comes at a higher computational cost.

Another drawback other automated methods have when dealing with overlapping dendrites is a reliance on correlation. Seed pixels for initialization can be chosen from the peaks of the correlation map, constructed by computing the average correlation of a pixel with its neighbors^43^. However, thin dendritic segments a few pixels wide may skew towards low correlation values and be systematically missed compared to wider somatic ROIs. Furthermore, when so many dendrites cross a given region (Fig. 1), the activity of those pixels will be a combination of all dendrites, which may obscure correlations within individual branches. d-NMF does not use correlation to initialize ROIs *per se*, but rather detects instances of synchronous activity across neighboring pixels to define initial spatial estimates (Fig. 2). This approach works in our densely labeled datasets because soma and dendrites tended to be sparsely active in time, such that only a few ROIs are active on any given frame. This mirrors the method by which manual segmentation is done, and allows identification of ROIs that may overlap with many others (Fig. 3).

Different aspects of d-NMF could be used in conjunction with pre-existing methods. Other segmentation software could be initialized with the initial ROI cores or the final ROIs obtained after de-mixing. The Fitness Trace (Fig. 4) could be used to screen any activity trace regardless of the method used to obtain it. Ultimately, different tools may be better suited to different data sets, so it is worthwhile for investigators to be able to combine the strengths of all available approaches.

Large population recordings allow the use of fewer animals to record from a given number of neurons, saving on time and resources. For characterizing single dendrite properties, this is particularly advantageous if a relatively small percentage of units show tuning to a given stimulus or behavioral feature. This is the case in CA3, where only ^~^20% of dendrites or soma show spatial selectivity (Fig. 5). Methods of inducing selectivity through artificial means^44,45^ can somewhat circumvent this, but the effects of these manipulations may be limited or constrained^46^. Larger population data not only adds more power to the analysis but also provide insights into functional heterogeneity and dynamics^47–49^. Simultaneous recording of multiple dendrites in the same animal provides within-subject controls on the effect of behavior on dendritic activity, and large enough population recording enables analysis of population-level stability (Fig. 6) and decoding of behavior (Fig. 7)^26,27,50^.

Long-term recordings of large populations of neurons have revealed changing neural representations over time, a phenomenon termed representational drift^51,52^. This has been documented in the hippocampus^53^ as well as several other brain areas^54–59^. Previous studies have shown that population responses of areas CA1 and CA3 of the hippocampus are particularly unstable across several days^42,53^, though other regions of the hippocampal circuit are more stable, including the dentate gyrus^42^ and entorhinal cortex^60^. Our observation of low stability across days in CA3 cell bodies is consistent with these observations. However, the different levels of stability in apical and basal dendrites (Figs. 6, 7) suggest that representational drift may not be homogenous across compartments of individual neurons.

Our results demonstrate that both apical and basal dendrites show spatial selectivity on par with nearby soma (Fig. 5), and the apical dendritic signal is more stable than the somatic or basal dendritic signal (Fig. 6). This suggests an interesting arrangement where the input arriving on to apical dendrites is stable across days yet the basal input changes from day to day. This may be related to the varying functionality of CA3 as performing pattern completion or pattern separation^61–65^. Such a switch may be the result of the network biasing propagation of inputs from apical or basal dendrites. A similar mechanism may be at work in the neocortex, where anatomy^66–68^ and theory^69–71^ suggests that bottom-up input arrives onto the basal dendrites while top-down input arrives on to the apical dendrites, providing a spatial segregation matching the functional segregation.

What could drive the difference in stability across dendritic compartments? These properties may be inherited from their upstream sources. Both entorhinal cortex and dentate gyrus, which target the apical dendrites of CA3, show a high degree of stability^42,60^. In contrast, the basal dendrites primarily receive recurrent excitation from CA3 soma, which themselves are largely unstable across days. Apical dendrites may also be more stable due to integrating signals from several different inputs. Conjunctive activity of entorhinal cortex, dentate gyrus, and recurrent collaterals may trigger supra-linearities such as dendritic spikes^7,18^, triggering long-term synaptic plasticity^16,18,44,72,73^ which could contribute to increased stability^21^. Differences in task demands also affect representational stability of soma in CA1^50^, so the stability of dendritic compartments under different behavioral tasks should be explored in the future.

It is also important to consider the contribution of forward-propagating versus back-propagating signals in defining dendritic activity. Basal dendrites are electrotonically close to the soma, so their activity may be largely back-propagated. However, the lower calcium transient rates in the basal dendrites suggest this propagation may not be entirely faithful. Basal dendrites had smaller place fields than soma, which may be a result of decreased event rate or could reflect more selective firing within a single session. Distal apical dendrites may enjoy a larger degree of independence because back-propagating action potentials often decay with distance^74^. Using calcium signals as an indicator of across-compartment correlation should be done with caution though, as calcium signals may reflect large bursts of activity more robustly than single action potentials^19^. Future studies using voltage-sensitive indicators or more sensitive calcium indicators in large populations should provide higher temporal resolution into these questions.

This study examined the activity of ensembles of apical dendrites, soma, and basal dendrites separately. A more in-depth investigation of input-output transformations at a sub-cellular level would require simultaneous measurement of connected dendrites and soma. By comparing the tuning properties of the apical dendrite, soma, and basal dendrite of the same neuron within densely labeled fields of view, one could learn about branch-specific^6,22,25,75^ versus branch prevalent activity^21,23,76^ and soma-dendrite coupling dynamics while sampling large populations. Such information has so far been limited to very sparse preparations, which may overlook cellular heterogeneity within regions^47–49^.

Our approach already merges together ROIs of neighboring dendrites and soma which have substantial spatial overlap and highly correlated activity. Such ROIs obtained from merging the soma and dendrites indicate a high degree of coupling between the soma and its proximal dendritic branches, as would be expected from the diffusion of calcium over short distances. But the possibility of dendrites exhibiting semi-independent activity^6,22,25,75^ from their cell bodies poses a challenge in linking ROIs based on correlations alone. Furthermore, many distal dendrites are connected to soma that are not in the focal plane, which could only be resolved with multi-planar imaging. These issues of connecting dendrites with parent soma will be important problems to address in future studies.

We developed and tested these tools primarily using viral expression of GCaMP6f. In principle, our approaches should work with different genetically encoded calcium indicators^77^ or voltage indicators^78,79^. These different indicators have varying signal-to-noise ratios and kinetics, so a reasonable parameter search may be necessary to obtain optimal performance of our tools. The rapid kinetics of voltage indicators with respect to the speed of propagation within dendrites may also result in the identification of finer-grained ROIs, as an assumption of our mathematical model is that the signal in a given ROI is uniform.

The methods presented here enable investigation into dendrites and soma, but are broadly applicable to any cell or cell process exhibiting dynamic fluorescence activity, including axons^18,80,81^ and astrocytes^82,83^. Any contiguous region of interest is identifiable using our core-finding approach. In our datasets we simultaneously detect soma, dendrites, and even putative axons without needing to change parameters. Characterizing the activity of afferent axons to a field of view is another tool in the circuit-mapper’s toolbox, providing a measure of the input to a brain region. Clever intersectional genetic approaches should be able to provide access to axonal input, dendritic processing, and somatic output in a single preparation, and our tools will facilitate the rigorous analysis of such datasets.

## Materials and Methods

### Animals

All experiments were conducted in accordance with the National Institute of Health guidelines and with the approval of the New York University School of Medicine Institutional Animal Care and Use Committee (IACUC). Imaging experiments used C57BL/6J mice as well as Ai9, Ai14, SST-Cre, and PV-Cre transgenic mice on a C57BL/6J background, from both sexes, 15–25 weeks old.

### Cranial window surgery

Surgery procedures were similar as described previously^8^. Mice were anesthetized using isoflurane (1.5–2.5%) and a 0.5 mm hole was drilled in the skull above dorsal area CA3 of hippocampus (1.6mm lateral, and 1.4 mm caudal of Bregma). For densely expressing preparations, injections of AAV1.CamKII. GCaMP6f.WPRE.SV40 (commercially generated at Penn Vector Core, titer: 2.76×10^13^ GC/ml, 23 nL per site) were made at two sites, two depth levels each (1.5 mm lateral, 1.3 mm caudal of Bregma; 1.7 mm lateral, 1.5 mm caudal of Bregma; depths of 1.8 and 2 mm below the dural surface). For sparser expression, we used a mixture of AAV1.CamKII-Cre (commercially generated at Penn Vector Core, diluted with ACSF) and AAV1.Syn.Flex.GCaMP6f.WPRE.SV40 or AAV1.Syn.Flex.jGCaMP7b.WPRE (commercially generated at Penn Vector Core) at 23 nL per site, at different ratios^8^ (see Fig. S1 for specific titers). After injection, a 3 mm craniotomy was made with the injection site at the center. The skull was removed, and a vacuum system was used to gently remove the overlying cortex and external capsule. Ice-cold ACSF was used to irrigate the area throughout the duration of the procedure. A cranial window (3 mm diameter, 1.7 mm length stainless steel cannula attached to 3 mm diameter glass coverslip) was then implanted over the area. The window was sealed to the skull using Vetbond, and a custom designed 3D- printed plastic headpost was cemented over the skull.

### Two-photon imaging

We used the same imaging system as described previously^8,50,84,85^. Mice were head-fixed on a treadmill belt and trained to run for 5% sucrose water delivered at random locations. *In vivo* two-photon imaging was performed using a dual galvanometric and resonant laser scanning two-photon microscope (Ultima, Bruker), coupled to a tunable Ti:Sapphire laser (MaiTai eHP DeepSee, Spectraphysics) pulsed at a 80 MHz repetition rates and <70 fs pulse width along with dispersion compensation. GCaMP fluorophore was excited at 920 nm, using a resonant scanning X- galvanometer (8 kHz) paired with a 6 mm standard scanning Y-galvanometer. The scanning system was mounted on movable objective Ultima microscope, equipped with an orbital nosepiece coupled to a 16X, 0.8NA, 3 mm water immersion objective (Nikon) and a piezo drive for angled imaging and ultrafast volumetric scanning. Imaging was performed at a scan speed of 29 fps, using 512×512 frame size (1.085 mm/pixel resolution). Fluorescence signal was detected using high-sensitivity GaAsP photomultiplier tubes (model 7422PA-40 PMTs, Hamamatsu). Recording sessions were ^~^10 minutes long, yielding datasets of ^~^10 GB.

### Head-fixed spatial navigation

Mice were trained to run head-fixed on a 2-meter linear treadmill belt similar to that described previously^18,50^. Sugar-water (5% sucrose) rewards were available from a lick spout in front of the mouse at random positions uniformly distributed on the track. The position of the mouse was measured using an optical rotary encoder (S5–720, US Digital). Lap onset was detected by reading RFID tags with an RFID reader mounted below the animal (ID-20LA, SparkFun Electronics). Behavioral programs were controlled with an Arduino Mega 2560 microcontroller. All behavioral data was acquired at a sampling rate of 10 kHz synchronized to the two-photon imaging. Mice ran on the same treadmill belt for 3 days to become acclimated to the textures and cues. The following days (Days 4–7) were designated as “familiar days” for the purposes of stability analysis. We always compared activity across consecutive familiar days for each animal. Taking data recording and template matching artifacts into account, Days 5 & 6 were used for all animals, except for two for which we compared Days 6 & 7 for one mouse and Days 4 & 5 for another.

### Statistics

Unless otherwise stated, all data are reported as the median and 95% confidence interval of the median, in the form M, [L, U], where M is the median, L is the lower bound of the 95% confidence interval, and U is the upper bound of the 95% confidence interval. The confidence interval was found through a resampling procedure previously described^6^.

All statistical comparisons were done using a Wilcoxon sign-rank (for paired data) or rank-sum (for unpaired data) unless otherwise specified (See section Subsampled Decoder).

All analyses were performed using custom-written codes in MATLAB R2018b (MathWorks).

### d-NMF

#### Pre-processsing

Image stacks were motion corrected for XY-motion using NoRMCorre^86^, and temporally downsampled by a factor of 20, resulting in image stacks with a time resolution of ^~^1.5 Hz. Each downsampled frame was computed as the mean of the surrounding frames, such that frame N of the downsampled image stack was computed from frames (N-1)*20+1 to N*20 of the original stack. These values were rounded and stored as unsigned 16-bit integers.

#### Division into Patches

Image stacks were divided up into patches of size 64×64, with an overlap of 8 pixels, yielding 81 patches.

#### ROI Core Detection

Within each patch, the image sequence was first de-trended and transformed into ΔF/F values by subtracting and dividing by the minimum value in a 45 frame (30 second) moving window. The threshold for a pixel to be “active” was set to be 3 times the median ΔF/F for each pixel. The thresholded binary image stack was then passed through a median filter of size 3 in the time dimension. Connected components of active pixels were then detected in the resulting 3-dimensional (X-Y-Time) image stack. Components with a minimum membership size of 30 pixels were projected into 2 (X-Y) dimensions. Those projected components with a minimum size of 15 pixels were kept, and the rest discarded. See Algorithm 1.

Components were then merged based on the Jaccard index (size of the intersection divided by the size of the union) between components, with a merge threshold of 0.5. Note that the same ROI active in distinct time spans will be detected multiple times in the previous step. Such ROIs are merged in this overlap step. Pixel values of each component are then normalized to sum to 1. Two additional background ROIs are included to capture local neuropil activity. Two extra ROIs are included, one composed of all pixels not belonging to any other ROI, and the other composed of all pixels in the area being considered. These are included to model background neuropil fluorescence.

#### Refinement and Activity Extraction

The following steps are standard fitting procedure for constrained non-negative matrix factorization^28,32^ (See Algorithm 2). The following steps are performed on the original image sequence patch, without de-trending or ΔF/F transformation. An image patch with t time steps and a d × d pixel size is stored as a matrix ***Y*** ∈ *R*^*d*×*d*×*t*^. This is reshaped into the matrix ${\text{Y}}^{*}\in R^{d^{2}\times t}$. The objective function is the following:

$$\underset{A,C}{\text{argmin}}\|Y-AC{\|}_{F}^{2}+\eta \|C{\|}_{F}^{2}+\beta \|\text{sum}(A){\|}^{2};A\geq 0,C\geq 0$$

where ‖∙‖_*F*_ denotes the Frobenius norm. For k components, $\text{A}\in R^{d^{2}\times k}$ represents the spatial footprints (ROIs), and ***C*** ∈ *R*^*k*×*t*^ represents the time-varying activity for each ROI. *η* and *β* are regularization terms to enforce sparsity of the temporal components (***C***) and spatial components (***A***), respectively. For results presented in this manuscript, *η* = 0.01, and *β* = 0.5.

The algorithm then iterates over solving for ***C*** and ***A*** while enforcing non-negativity. The optimal solutions for ***C*** and ***A*** are as follows:

$$C^{*}=\text{max}\left(\left(A^{⊤}A+\eta I\right){\left(Y^{⊤}A\right)}^{†},0\right)\;A^{*}=\text{max}\left(YC^{⊤}{\left(CC^{⊤}+\beta 1\right)}^{†},0\right),$$

Where **I** represents the identify matrix, and **1** represents a matrix with each element equal to 1. At each iteration *n*, C and A are then updated from their current values with a learning rate of 0.5:

$$C_{\text{n}+1}=0.5C_{\text{n}}+0.5C^{*}\;A_{\text{n}+1}=0.5A_{\text{n}}+0.5A^{*}$$

After convergence, the resulting ROIs are passed through a 2-dimensional median filter of size 3×3, and the top 10% of pixel values are kept. The resulting ROIs are split into contiguous components, and the time courses are calculated one final time by updating ***C***. Only ROIs with a minimum size of 30 pixels are included.

#### Combining Results from Patches

After all image patches are processed, the resulting ROIs are evaluated to be merged. The temporal traces are detrended by subtracting the minimal value in a rolling 45 frame (30 second) window. For two ROIs to be merged, their temporal components must have a correlation coefficient above 0.9, and they must share at least 1 pixel in membership. The spatial and temporal components of merged ROIs are then updated according to Algorithm 3 (below).

### ROI Comparisons

#### Manual Labeling

Ground truth labeling data was generated by human experts who manually labeled ROIs using the software program FIJI^87^. The motion-corrected, temporally downsampled image stacks were loaded in to memory, and inspected frame-by-frame for neural segments showing synchronous activity. Dendritic branches were split up into multiple ROIs at branch points. Additionally, labelers were instructed to break up long dendritic branches into smaller ROIs about the length of the soma (approximately 30 pixels).

#### Skewness Classification

The output of d-NMF contains a mixture of valid and invalid ROIs. To automatically classify ROIs as valid or invalid, we computed the skewness of the extracted temporal traces after de-trending with a sliding window of 45 time points (30 seconds). For ground truth, human labelers manually screened all extracted ROIs and labeled them as ‘valid’ or ‘invalid’ based on morphology. Using skewness as a threshold value, we quantified the performance of the classifier using two metrics: the area under the ROC curve plotting the True Positive Rate versus the False Positive Rate, and the Jaccard Index, computed as (TP/(TP+FP+FN)), where TP is the count of True Positives, FP is the count of False Positives, and FN is the count of False Negatives (Fig. 3c–d).

#### Signal to noise ratio

We also tested the efficacy of the signal-to-noise ratio as a classifier threshold (Extended Data Fig. 3). For a detrended calcium trace, the signal-to-noise ratio was defined as SNR = P/M Where P is the 99.9^th^ percentile of the trace, and M is the median absolute deviation of the trace (median(|x – median(x)|)). We defined SNR this way to be robust to noise and to be able to define the noise levels without contamination from large transients.

#### CaImAn Parameters

The python implementation of CaImAn^28^ was run on the motion corrected, downsampled image stacks using default parameters using the “sparse_nmf” initialization procedure. Patch sizes and overlaps were chosen to be 64 × 64 and 8 pixels to match those used by d-NMF. K = 15 components per patch were chosen to be estimated. Only those ROIs labeled as “valid” were used for analyses (Fig. 3).

#### suite2p Parameters

Similarly, suite2p was run on the motion corrected, downsampled image stacks using pre-configured parameters for dendrites and axons. All labeled ROIs were used for analysis (Fig. 3).

#### F1 score for model comparison

The output of d-NMF, CNMF, and suite2p were compared to manually labeled data in a pixel-wise fashion. The metric used was the F1 score, calculated as

$$F1=2*\frac{\text{Precision}*\text{Recall}}{\text{Precision}+\text{Recall}}$$

This measure is a combination of recall (True Positive Rate, TP/(TP+FN)) and precision (Positive Predictive Value, TP/(TP+FP)), as human-labeled datasets may have incomplete labeling, which would yield poor estimates of false positives.

### Fitness Trace

To generate ground truth data to evaluate the Fitness Trace (Fig. 4), 90 ROIs generated from d-NMF (30 apical dendrites, 30 soma, and 30 basal dendrites, when possible) were chosen at random from 9 FOVs. Their activity traces were re-estimated by taking a weighted average across the ROI, which was then de-trended using a rolling minimum of 45 frames, then z-scored. Putative transients were identified as contiguous time bins where the z-scored trace exceeded 2 standard deviations. These event detection criteria was meant to be permissive, as it was not performed on the de-mixed data, so false transients would likely be present. This was by design, to contain examples of valid and invalid transients. Transients were then manually reviewed and classified as valid or invalid using a previously published graphical user interface^37^.

The image stack was de-trended and transformed into ΔF/F values, then z-scored across time. For a given ROI, a square bounding box was defined around all non-zero values with a padding of 3 pixels in each dimension. The Fitness Trace for a given ROI at time *t* was defined as the correlation coefficient between the z-scored image stack at time t and the ROI, calculated only using the pixels in that bounding box.

#### Transient Detection Methods

To describe the different detection methods (Fig. 4, Extended Data Fig. 4), it is useful to define two types of activity traces. The first is the activity trace arrived at through d-NMF, that is, a signal designed to be de-mixed from overlapping sources; we will denote this as De-mixed ΔF/F. The second is a “simple” signal obtained through taking a weighted average across all of the pixels of an ROI. We will denote this as Simple ΔF/F. Unless otherwise noted, activity traces are all de-trended by subtracting the rolling minimum across 45 frames, then z-scored.

**Table:** 

Name	2z	Opt	Fit	SEUDO	D_2z_	D_Opt_	D_Fit_	D_SEUDO_
**Primary Trace**	Simple ΔF/F	Simple ΔF/F	Simple ΔF/F	Simple ΔF/F	De-mixed ΔF/F	De-mixed ΔF/F	De-mixed ΔF/F	De-mixed ΔF/F
**Threshold**	2z	Variable	Variable	Variable	2z	Variable	Variable	Variable
**Secondary Trace**	None	None	Fitness	SEUDO^37^	None	None	Fitness	SEUDO
**Threshold**	N/A	N/A	Variable	Variable	N/A	N/A	Variable	Variable

### Spatial Tuning Properties

Single session analyses (Fig. 5, Extended Data Figs. 5–8) were performed on data from Familiar Day 1. Behavioral data was first downsampled to match the frame rate of imaging. Only data during movement (speed > 2 cm/s) was used for all spatial analyses. To construct firing rate maps, the track was divided into 40 equally spaced bins of width 5 cm. Occupancy and transient event count were computed, circularly smoothed with a Gaussian smoothing kernel of width 5cm, and then divided to compute the rate.

ROIs were manually classified as apical dendrites, soma, or basal dendrites based on position in the field of view. ROIs encompassing both a soma and one or more dendritic branches were classified as soma. In all other instances we treat apical dendrites, soma, and basal dendrites as independent populations, without assigning parent soma to individual dendrites. Thus, when we refer to apical dendrites or basal dendrites, we are referring to dendrites which are some distance from their parent cell body, which is often not visible in the same field of view.

We used two measures to quantify the degree of tuning of each rate map. Maps were divided into 40 bins as described above. Spatial information content^88^ was computed as

$$\text{Info}\;\text{Content}=\sum_{i=1}^{N}P_{i}R_{i}{\text{log}}_{2}R_{i}$$

Where P_i_ is the normalized occupancy in the *i*th bin, such that the sum across all P_i_ = 1, and R_i_ is the normalized value of the rate map in the *i*th bin, such that the sum across all R_i_ = 1. Sparsity was computed as

$$\text{Sparsity}=1-\frac{{\left(\sum_{i=1}^{N}R_{i}\right)}^{2}}{N\sum_{i=1}^{N}R_{i}^{2}}$$

Where R_i_ is the value of the rate map in the *i*th bin.

Each ROI served as its own control to determine statistical significance of spatial tuning. For each ROI, the transient times were shifted by n time bins, for n = −250 to 250. The information content at a shift of 0 was then normalized to the 99^th^ percentile of information content at non-0 shift. A normalized information content value greater than 1 indicated significant tuning. Additionally, a minimum of 4 transients must have been detected for an ROI to be called significantly tuned.

#### Tuning Parametrization

Tuning curves were parametrized as a mixture of Von Mises functions, as described previously^89^. Rate maps were circularly smoothed with a sigma of 15 cm (3 bins), and the curve was fit by increasing the number of Von Mises functions until the residuals were below 25% of the maximum value of the original rate map. Place field width was defined as the width of a fitted component at 50% of the component’s amplitude (i.e. full width at half max). For ROIs with multiple place fields, their width was defined as the mean width of all place fields.

### Quantifying Overlap

We defined two ROIs as overlapping if they shared at least 8 pixels and had a correlation coefficient between their activity traces less than 0.75. This avoids inflating the overlap amount from ROIs that are part of the same dendritic tree.

### Stability

Within-day stability (Fig. 6) was assessed by splitting the data from Familiar Day 1 in half. Rate maps were re-estimated for each ROI using only the data in each session half. Tuning curve (TC) correlation was defined as the correlation coefficient between the tuning curves for each half. This measure was computed for each ROI and then averaged to obtain a single value for the session. To be included in the calculation for TC correlation, an ROI must have had a minimum of 4 calcium transients and be significantly tuned across the entire session. Population vector (PV) correlation was computed by constructing the rate estimates of all ROIs for a given position bin in the first half and taking the correlation coefficient between that vector and the corresponding vector in the second half. This yields a value for each position bin, which was then averaged to obtain a single value for a session. To be included in the calculation for PV correlation, an ROI must have had a minimum of 4 calcium transients, with no criteria for significant tuning.

To quantify across-day stability (Fig. 6, Extended Data Figs. 9–10), image stacks from Familiar Days 1 and 2 were stitched into a single image stack and motion corrected together. d-NMF was then run on the combined image stack to obtain ROIs that spanned both sessions. Stability analysis was then performed as for within-day estimates. To be included in across-day stability analyses, an ROI must have had at least 4 detected calcium transients in both days and significantly tuned in at least one day. The activity criterion ensured that correlation estimates were not skewed by ROIs that were not visible on one of the recording days.

#### Structural Stability

To verify that differences in tuning stability were not due to the inability to track ROIs across days, we additionally quantified the structural stability of ROIs (Extended Data Fig. 10). For each ROI we took the average of frames at which significant calcium transients were detected for Familiar Days 1 and 2. The structural stability was defined as the correlation coefficient between these averages, using only data within the modified boundary of the ROI. The modified boundary of an ROI was defined by first thresholding the ROI above 20% of its maximum pixel intensity, then dilating the resulting mask by one pixel.

### Population Vector Decoding

To test the impact of increased across-day stability in apical dendrites, we performed population vector decoding in a manner similar to that described previously^50^ (Fig. 7). A separate decoder was constructed for each FOV for each of apical dendrites, soma, and basal dendrites. Template tuning curves for each ROI were constructed as above. For within-day decoding, data from the first half of Familiar Day 1 was used to define the template. For across-day decoding, all data from Familiar Day 1 was used to define the template. As above for defining Population Vector correlations, only ROIs that were active in both days and significantly tuned in at least one day were included for decoding.

Time-varying rate vectors for each ROI were constructed using data from either the second half of Familiar Day 1 (within-day decoding) or all data from Familiar Day 2 (across-day decoding), using 667 ms bins smoothed with a Gaussian smoothing kernel with σ = 3.3 seconds. For each time point in the decoded portion of the data, the decoded position was the position corresponding to the highest correlation with the template matrix. Any time points with zero activity across all relevant ROIs had undefined correlation with any position. The most recently decoded position was copied in to those undefined frames.

Decoding error was defined as the median absolute error between decoded position and actual position, defined circularly so the beginning of the track (position 0 cm) and the end of the track (position 200 cm) were 0 cm away.

#### Subsampled decoder

The performance of a population vector decoder is highly dependent on the number of units used to construct it^50^. To control for variation in the number of ROIs across compartments, we constructed additional population vector decoders using subsets of eligible ROIs (Fig. 7d–e). For each session and compartment type (apical, soma, basal), we randomly selected (without replacement) 5, 15, 25, 35, 45, 55, or 65 ROIs if available to decode position and record decoding error. This process was repeated 100 times and the median value across the 100 iterations was used as the decoding error of the subsampled classifier.

Statistical significance of decoding for subsampled decoders was assessed using a two-way ANOVA with the number of ROIs as a continuous predictor, the ROI type (Apical, Soma, or Basal) as a categorical predictor, and the decoding error as the dependent variable. P-Values reported in Fig. 7 are the p-values for an interaction effect between the number of ROIs and the ROI type, performed in a pairwise (Apical vs Soma, Apical vs Basal, Soma vs Basal) manner.


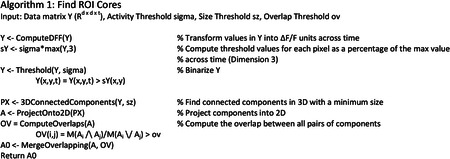



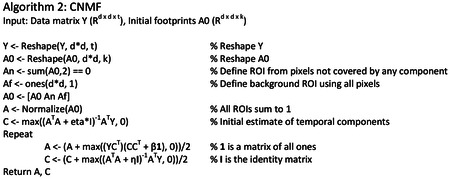



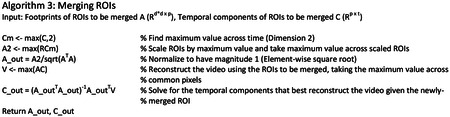


## Extended Data

## Figures and Tables

**Table 1 |:** Statistics for Extended Data Fig. 4.

Panel	Measure	Fit	Opt	2z	Fit vs Opt	Fit vs 2z	Opt vs 2z
**c**	Jaccard, d-NMF	0.65, [0.62, 0.73]	0.63, [0.59, 0.68]	0.50, [0.41, 0.54]	p=0.01	p=0.004	p=0.004
**c**	Jaccard, Simple	0.64, [0.59, 0.76]	0.50, [0.47, 0.55]	0.44, [0.36, 0.48]	p=0.008	p=0.004	p=0.01
**c**	D_Fit_ vs Fit: p=0.50						
**d**	Rate, d-NMF	0.48, [0.32, 0.55]	0.44, [0.37, 0.51]	0.85, [0.74, 0.96]	p=0.10	p=0.004	p=0.004
**d**	Rate, Simple	0.47, [0.30, 0.58]	0.56, [0.45, 0.62]	1.07, [0.84, 1.13]	p=0.004	p=0.004	p=0.004
**d**	D_Fit_ vs Fit: p=0.13						
		D_Fit_	D_SEUDO_	Comparison	Fit	SEUDO	Comparison
**e**	Jaccard	0.65, [0.62, 0.73]	0.63, [0.59, 0.68]	p=0.02	0.64, [0.59, 0.76]	0.62, [0.48, 0.69]	p=0.008
**F**	Rate	0.48, [0.32, 0.55]	0.44, [0.36, 0.51]	p=0.07	0.47, [0.30, 0.58]	0.52, [0.32, 0.61]	p=0.30

**Table 2 |:** Statistics for Extended Data Fig. 5a–f.

Panel	Measure	A	S	B	A vs S	A vs B	S vs B
**a**	# Fields (D_Fit_)	1.07, [1.04, 1.12]	1.01, [1.00, 1.09]	1.03, [1.00, 1.06]	p=0.38	p=0.055	p=1
	# Fields (2z)	1.21, [1.18, 1.34]	1.23, [1.13, 1.30]	1.25, [1.20, 1.36]	p=0.64	p=0.46	p=0.64
**b**	Info Content – All (D_Fit_)	2.12, [1.78, 2.28]	2.06, [1.91, 2.37]	2.16, [2.05, 2.22]	p=0.95	p=0.38	p=0.2
	Info Content – All (2z)	0.96, [0.88, 1.28]	1.27, [1.16, 1.42]	0.90, [0.74, 0.97]	p=0.023	p=0.11	p=0.0078
**c**	Sparsity – All (D_Fit_)	0.79, [0.74, 0.81]	0.78, [0.76, 0.82]	0.79, [0.75, 0.81]	p=1	p=0.25	p=0.38
	Sparsity – All (2z)	0.56, [0.52, 0.64]	0.63, [0.61, 0.67]	0.54, [0.48, 0.57]	p=0.023	p=0.055	p=0.0078
**d**	Norm. Info Content – All (D_Fit_)	0.86, [0.81, 0.94]	0.85, [0.81, 0.91]	0.85, [0.80, 0.91]	p=0.95	p=0.84	p=0.74
	Norm. Info Content – All (2z)	0.83, [0.73, 0.96]	0.83, [0.75, 0.87]	0.83, [0.70, 0.94]	p=0.64	p=0.95	p=1
**e**	Info Content – Tuned (D_Fit_)	2.33, [2.10, 2.64]	2.27, [2.15, 2.43]	2.58, [2.48, 2.66]	p=0.95	p=0.11	p=0.016
	Info Content – Tuned (2z)	1.33, [1.18, 1.53]	1.65, [1.50, 1.91]	1.17, [1.11, 1.23]	p=0.078	p=0.2	p=0.055
**f**	Sparsity – Tuned (D_Fit_)	0.82, [0.79, 0.86]	0.81, [0.80, 0.84]	0.85, [0.84, 0.86]	p=0.95	p=0.078	p=0.016
	Sparsity – Tuned (2z)	0.66, [0.63, 0.70]	0.72, [0.69, 0.76]	0.64, [0.63, 0.65]	p=0.2	p=0.25	p=0.11

**Table 3 |:** Statistics for Extended Data Fig. 5g–o.

Panel	Measure	D_Fit_	2z	Comparison
**g**	Rate (A)	0.52, [0.41, 0.63]	1.31, [1.17, 1.49]	p=0.0078
	Rate (S)	0.51, [0.42, 0.62]	1.09, [0.93, 1.22]	p=0.0078
	Rate (B)	0.40, [0.35, 0.45]	1.53, [1.44, 1.61]	p=0.0078
**h**	% Tuned (A)	20.7, [13.4, 28.5]	23.9, [14.7, 34.6]	p=0.22
	% Tuned (S)	19.8, [12.7, 27.6]	22.1, [13.5, 32.1]	p=0.16
	% Tuned (B)	17.7, [11.5, 24.1]	24.5, [14.1, 36.5]	p=0.15
**i**	Place Field Width (A)	23.2, [21.6, 24.5]	31.0, [27.7, 35.2]	p=0.0078
	Place Field Width (S)	24.2, [21.9, 26.6]	26.9, [25.1, 28.8]	p=0.055
	Place Field Width (B)	20.7, [19.1, 22.3]	28.7, [27.0, 31.1]	p=0.0078
**j**	# Fields (A)	1.08, [1.04, 1.11]	1.23, [1.18, 1.29]	p=0.016
	# Fields (S)	1.04, [1.01, 1.08]	1.23, [1.16, 1.32]	p=0.0078
	# Fields (B)	1.03, [1.01, 1.04]	1.26, [1.20, 1.31]	p=0.0078
**k**	Info Content – All (A)	2.05, [1.87, 2.21]	1.07, [0.92, 1.24]	p=0.0078
	Info Content – All (S)	2.06, [1.87, 2.22]	1.25, [1.11, 1.36]	p=0.0078
	Info Content – All (B)	2.11, [1.98, 2.23]	0.91, [0.80, 1.04]	p=0.0078
**l**	Sparsity – All (A)	0.77, [0.74, 0.80]	0.58, [0.53, 0.62]	p=0.0078
	Sparsity – All (S)	0.77, [0.74, 0.80]	0.62, [0.59, 0.65]	p=0.0078
	Sparsity – All (B)	0.78, [0.76, 0.80]	0.53, [0.50, 0.57]	p=0.0078
**m**	Norm. Info Content – All (A)	0.86, [0.82, 0.91]	0.83, [0.77, 0.90]	p=0.15
	Norm. Info Content – All (S)	0.86, [0.81, 0.91]	0.84, [0.77, 0.91]	p=0.25
	Norm. Info Content – All (B)	0.85, [0.81, 0.90]	0.84, [0.76, 0.92]	p=0.64
**n**	Info Content – Tuned (A)	2.36, [2.17, 2.55]	1.35, [1.23, 1.48]	p=0.0078
	Info Content – Tuned (S)	2.29, [2.20, 2.37]	1.59, [1.34, 1.78]	p=0.0078
	Info Content – Tuned (B)	2.56, [2.48, 2.63]	1.23, [1.14, 1.37]	p=0.0078
**o**	Sparsity – Tuned (A)	0.82, [0.80, 0.84]	0.66, [0.64, 0.69]	p=0.0078
	Sparsity – Tuned (S)	0.82, [0.80, 0.83]	0.70, [0.65, 0.74]	p=0.0078
	Sparsity – Tuned (B)	0.85, [0.84, 0.86]	0.64, [0.62, 0.67]	p=0.0078
